# Single cell transcriptomic analysis of murine lung development on hyperoxia-induced damage

**DOI:** 10.1038/s41467-021-21865-2

**Published:** 2021-03-10

**Authors:** Maria Hurskainen, Ivana Mižíková, David P. Cook, Noora Andersson, Chanèle Cyr-Depauw, Flore Lesage, Emmi Helle, Laurent Renesme, Robert P. Jankov, Markku Heikinheimo, Barbara C. Vanderhyden, Bernard Thébaud

**Affiliations:** 1 Sinclair Centre for Regenerative Medicine, Ottawa Hospital Research Institute, Ottawa, ON Canada; 2 Division of Pediatric Cardiology, New Children’s Hospital, Helsinki University Hospital and University of Helsinki, Helsinki, Finland; 3 Pediatric Research Center, New Children’s Hospital, University of Helsinki and Helsinki University Hospital, Helsinki, Finland; 4 Department of Cellular and Molecular Medicine, University of Ottawa, Ottawa, ON Canada; 5 Cancer Therapeutics Program, Ottawa Hospital Research Institute, Ottawa, ON Canada; 6 Research Programs unit, Systems Oncology, Faculty of Medicine, University of Helsinki, Helsinki, Finland; 7 Research Programs Unit, Stem Cells and Metabolism, University of Helsinki, Helsinki, Finland; 8 Department of Pediatrics, Children’s Hospital of Eastern Ontario (CHEO) and CHEO Research Institute, University of Ottawa, Ottawa, ON Canada; 9 Molecular Biomedicine Program, Children’s Hospital of Eastern Ontario Research Institute, Ottawa, ON Canada; 10 Department of Obstetrics and Gynecology, University of Ottawa/The Ottawa Hospital, Ottawa, ON Canada

**Keywords:** Sequencing, Cell biology, Mechanisms of disease, Molecular biology, Transcriptomics

## Abstract

During late lung development, alveolar and microvascular development is finalized to enable sufficient gas exchange. Impaired late lung development manifests as bronchopulmonary dysplasia (BPD) in preterm infants. Single-cell RNA sequencing (scRNA-seq) allows for assessment of complex cellular dynamics during biological processes, such as development. Here, we use MULTI-seq to generate scRNA-seq profiles of over 66,000 cells from 36 mice during normal or impaired lung development secondary to hyperoxia with validation of some of the findings in lungs from BPD patients. We observe dynamic populations of cells, including several rare cell types and putative progenitors. Hyperoxia exposure, which mimics the BPD phenotype, alters the composition of all cellular compartments, particularly alveolar epithelium, stromal fibroblasts, capillary endothelium and macrophage populations. Pathway analysis and predicted dynamic cellular crosstalk suggest inflammatory signaling as the main driver of hyperoxia-induced changes. Our data provides a single-cell view of cellular changes associated with late lung development in health and disease.

## Abstract (web summary)

It is unclear how changes in gene expression are induced by changes in oxygen levels during late lung development. Here, the authors provide data from MULTI-seq scRNAseq in mice showing exposure to higher oxygen levels affects cell fates, especially for alveolarisation, and define gene/cell signatures of impaired lung development under hyperoxia.

## Introduction

Late lung development is responsible for the formation of intricate structures enabling the exchange of inspired oxygen from the atmosphere and carbon dioxide from the blood, which is the primary function of the mammalian lung. This complex task of gas exchange is achieved in the smallest, most distal respiratory units of the lung (the alveoli) and occurs across a thin structure (0.2−2 μm) of the alveolo-capillary barrier covering a vast surface area of the lung (~75 m^2^). The formation of this complex structure is achieved via interconnected events of secondary septa formation and microvascular maturation during the period of late lung development. These processes are facilitated by temporarily and spatially coordinated crosstalks between multiple cell types in the lung microenvironment. In addition to gas exchange, the lung acts as an important immune barrier, requiring resident alveolar macrophages to transition toward a mature anti-inflammatory phenotype. However, the signals driving these processes and the landscape of resident cells during late lung development remain largely uncharacterized^1–3^.

In humans, impaired late lung development presents as bronchopulmonary dysplasia (BPD), the most common chronic lung disease in children. BPD is a multifactorial disease with some hereditary component^4,5^, occurring as a consequence of premature birth and the result of an aberrant reparative response to both antenatal and repetitive postnatal injury to the developing lungs^6^. In addition to impaired alveolar and microvascular formation, immune development of the lung is interrupted, leading to recurrent bacterial and viral respiratory infections. To mimic these injuries, rodent models of BPD utilize various levels of hyperoxia and/or other pro-inflammatory stimuli. Sustained exposure of neonatal mice to hyperoxia leads to a BPD-like lung phenotype, making it an ideal model to identify and study pivotal developmental steps during late lung development^7^.

The role of various cell types during late lung development has been extensively studied, establishing important functions for myofibroblasts in secondary septation, endothelial cell (EC) signaling in the processes of microvascular maturation and coordination of inflammatory cell signaling^7^. The abundance and identity of individual cell types are dynamic throughout lung development. Identification and classification of lung cells become even more complex under pathological conditions, in particular when the nature of the disease is heterogenous. Traditional methods to assess molecular characteristics of pathologies have depended on bulk measurements of protein or RNA, but given the heterogeneity of lung tissue and its dynamics during late development, these measurements are confounded by changes in cellular composition. As a result, changes in individual cell types cannot be identified. This is more problematic when responses are limited to rare populations, as these changes will be masked by the signal from more-abundant cell types. To circumvent these obstacles, we herein employed multiplexed single-cell RNA sequencing (scRNA-seq) to resolve changes in cellular composition and state during both normal and impaired late lung development.

Here, we report an extensive profiling of the cellular composition in the developing mouse lung by generating scRNA-seq profiles of 66,200 cells from 36 normally and aberrantly (O_2_-exposed) developing mouse lungs at three time points (P3, P7, and P14). We also validate some of our findings by fluorescent RNA in situ hybridization in lung samples from BPD patients. In this study, we observe greatly diverse and dynamic populations of cells. Hyperoxia exposure alters the phenotype of all major cell types, particularly capillary endothelium, stromal, and macrophage populations. Our data suggest that inflammatory activation is the major driver of the observed transcriptional changes in hyperoxia. In conclusion, we identify multiple cell-specific gene signatures, which provide a detailed cell and molecular atlas of normal and impaired postnatal lung development.

## Results

### Detailed map of cellular composition during normal and impaired late murine lung development

**Fig. 1:**
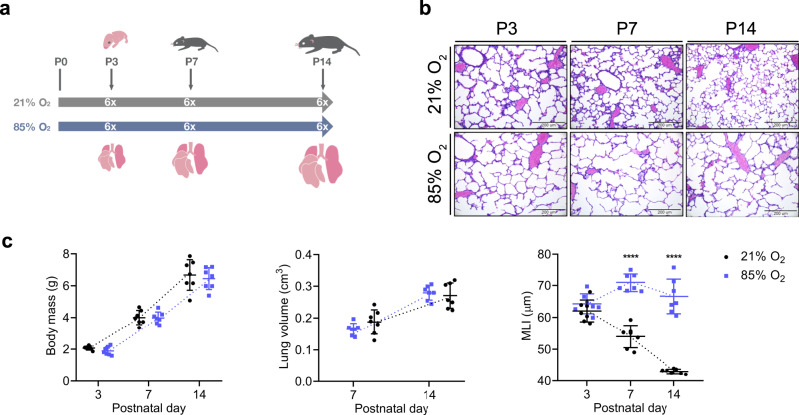
Exposure to hyperoxia induced an arrest in alveolarization in the developing mouse lung. **a** Mouse pups were exposed from day of birth to room air (21% O_2_, gray) or hyperoxia (85%O_2_, blue). A total of 36 lungs were harvested on postnatal days (P)3, 7, and 14. *n* = 6/group. **b** Representative histological sections from lungs developing in 21% O_2_ (black circles) or 85% O_2_ (purple squares) at P3, P7, and P14. Seven animals/group were evaluated. Scale bar = 200 µm. **c** Body weight was assessed at P3, P7, and P14. Lung volume was assessed by Archimedes principle at P7 and P14. Precise measurements at P3 were not possible due to the small size of the organ. Lung morphometry was quantified by the mean linear intercept (MLI) measurement. Data are presented as means ± SD. Statistical analyses were performed with GraphPad Prism 8.0. The presence of potential statistical outliers was determined by Grubbs’ test. Significance was evaluated by multiple unpaired Student’s *t-*test with Holm–Sidak correction. *P* values **** = <0.0001. *n* = 7 animals/group.

In order to create a comprehensive cellular map of the normal and impaired developing lung, we generated scRNA-seq profiles of 36 mice on postnatal days (P)3, 7, and 14 (Fig. 1a). Impaired lung development was induced by normobaric hyperoxia (85% O_2_) from day of birth (P0) to P14 (Fig. 1a–c) and was independent of body weight or lung volume (Fig. 1a–c). In order to capture diverse cell populations present in the lung, we optimized the single-cell preparation protocol by testing several digestion conditions as assessed by FACS and scRNA-seq analysis (Supplementary Fig. 1a, b). To evaluate the actual cell contribution in vivo prior to tissue digestion, we performed a stereological assessment of alveolar epithelial type 2 (AT2) cells. The number of AT2 cells in vivo was not impacted by hyperoxia as assessed by stereology, supporting the validity of our observations (Supplementary Fig. 1c; Supplementary Table 1). In total, we generated scRNA-seq expression profiles for 66,200 cells (∼11,033 cells/group) (Supplementary Fig. 1d). Single-cell suspensions from individual mice were multiplexed using MULTI-seq^8^ (Supplementary Fig. 1e, f). No major sex-dependent bias in cell distribution could be observed (Supplementary Fig. 1g, h).

**Fig. 2:**
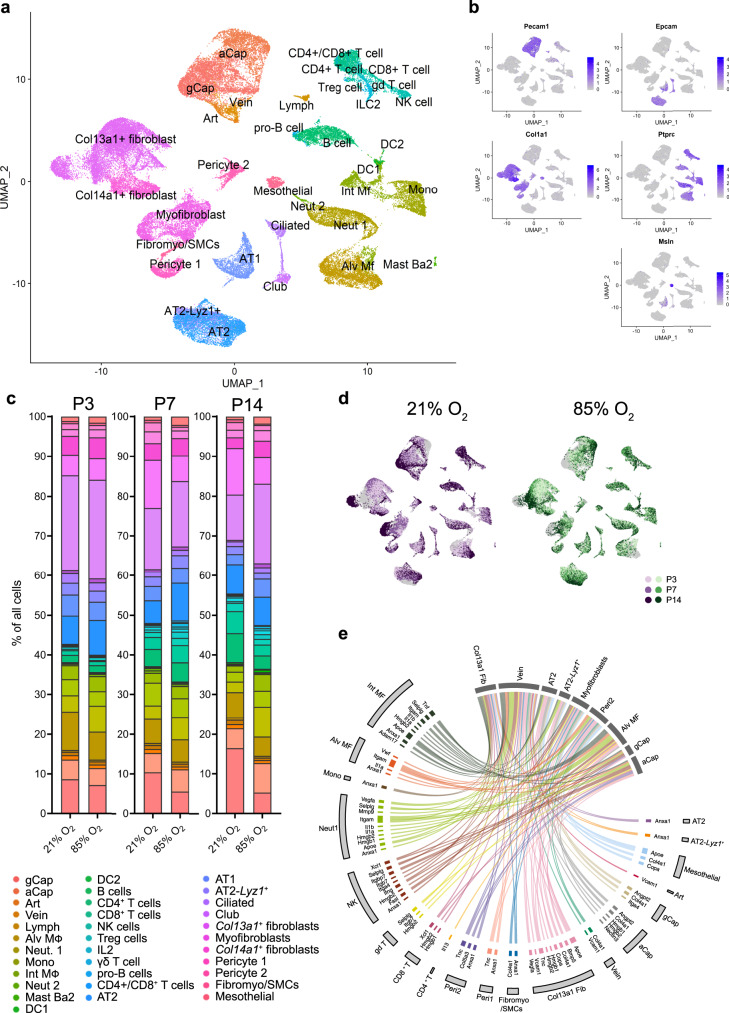
Map of cellular composition in normal and hyperoxia-impaired late murine lung development. **a** UMAP plot of all scRNA-seq data, showing a total of 34 distinct cell types that were identified. **b** UMAP plots showing expression levels for canonical markers of epithelial, mesenchymal, endothelial, immune, and mesothelial populations. The intensity of expression is indicated by purple coloring. **c** UMAP plots of normally (21% O_2_-exposed; left) and aberrantly (85% O_2_-exposed; right) developing lungs. Each cell is colored by mouse age as indicated by the legend. **d** Cluster distribution in lungs of normally and aberrantly developing mice at P3, P7, and P14. *n* = 6 animals/group. **e** Circos plot showing inferred cell communications. Cell types in the top right correspond to those with the largest changes in response to hyperoxia. These cell types are connected to the cell types expressing ligands predicted to promote this response. Ligands expressed by the same cell population are colored the same. Expression levels in UMAP plots are presented as log(TP10k + 1) values. Log(TP10k + 1) corresponds to log-transformed UMIs per 10k. Cell populations in **a** and **d** are colored as indicated by the legend in **d**.

Cells were clustered based on their expression profile, and cell types were annotated based on established cell markers available on LungMap^9^ (https://lungmap.net/), CellMarker^10^, The Human Protein Atlas^11^ (http://www.proteinatlas.org), and in the published literature (Fig. 2a; Supplementary Data 1). A total of 34 clusters were identified, corresponding to six major cell groups: epithelial, stromal, endothelial, myeloid, lymphoid, and mesothelial cells (Fig. 2b; Table 1; Supplementary Fig. 2a, b, Supplementary Movie 1). We observed dynamic changes in the cellular composition of normally developing lungs. Most changes occurred between P7 and P14. Impaired alveolar development induced by hyperoxia changed the cellular distribution of the lung at all time points (Fig. 2c, d; Supplementary Fig. 2a, b; Supplementary Data 2). The expression of BPD-associated genes in our data showed gene-specific distinct cellular expression patterns with the highest expression in capillary endothelial cells, macrophages and neutrophils, NK, mast and basophil and T cells, AT1 and AT2 cells, fibroblasts and mesothelial cells, many of which were indicated as most reactive to hyperoxia in our other data analysis (Supplementary Fig. 3). We then used the NicheNet tool^12^ to infer cellular communications specific to hyperoxia-associated gene expression patterns based on expression of ligands, receptors, associated pathway components, and genomic targets of these pathways (Fig. 2e). According to differentially expressed genes in hyperoxia samples, our cell communication inference indicated that the effects of hyperoxia were mediated mostly by inflammatory signals (Fig. 2e). Our signaling pathway and cell communication analysis suggests that hyperoxia initiates inflammation with activation of particular endothelial, epithelial, stromal and resident lung immune cell subpopulations promoting innate and adaptive immune responses, fibrosis and several pathways disturbing endothelial development and homeostasis.

**Table 1:** Identified cell populations.

Abbreviation	Cell type	Abbreviation	Cell type
gCap	General capillary endothelial cells	Treg cells	Regulatory T cells
aCap	Capillary endothelial cells - aerocytes	ILC2	Innate lymphoid cells 2
Art	Arterial endothelial cells	γδ T cells	γδ T cells
Vein	Venous endothelial cells	pro-B cells	B-cell progenitors
Lymph	Lymphatic endothelial cells	CD4 + /CD8 + T cells	CD4^+^/CD8^+^ T cells
Alv MΦ	Alveolar macrophages	AT2	Alveolar type 2 cells (*Lyz1*^-^)
Neut 1	Neutrophils 1	AT1	Alveolar type 1 cells
Mono	Monocytes	AT2-*Lyz1*^+^	Alveolar type 2 cells (*Lyz1*^+^)
Int MΦ	Interstitial macrophages	Ciliated	Ciliated cells
Neut 2	Neutrophils 2	Club	Club cells
Mast Ba2	Mast basophils 2	*Col13a1*^+^ fib	*Col13a1*^+^ fibroblasts
DC1	Dendritic cells 1	Myofib.	Myofibroblasts
DC2	Dendritic cells 2	*Col14a1*^+^ fib	*Col14a1*^+^ fibroblasts
B cells	B cells	Pericyte 1	Pericytes 1
CD4^+^ T cells	CD4^+^ T cells	Pericyte 2	Pericytes 2
CD8^+^ T cells	CD8^+^ T cells	Fibromyo./SMCs	Fibromyocytes/Smooth muscle cells
NK cells	NK cells	Mesothelial	Mesothelial cells

### Hyperoxia alters AT2 cell populations during late murine lung development

**Fig. 3:**
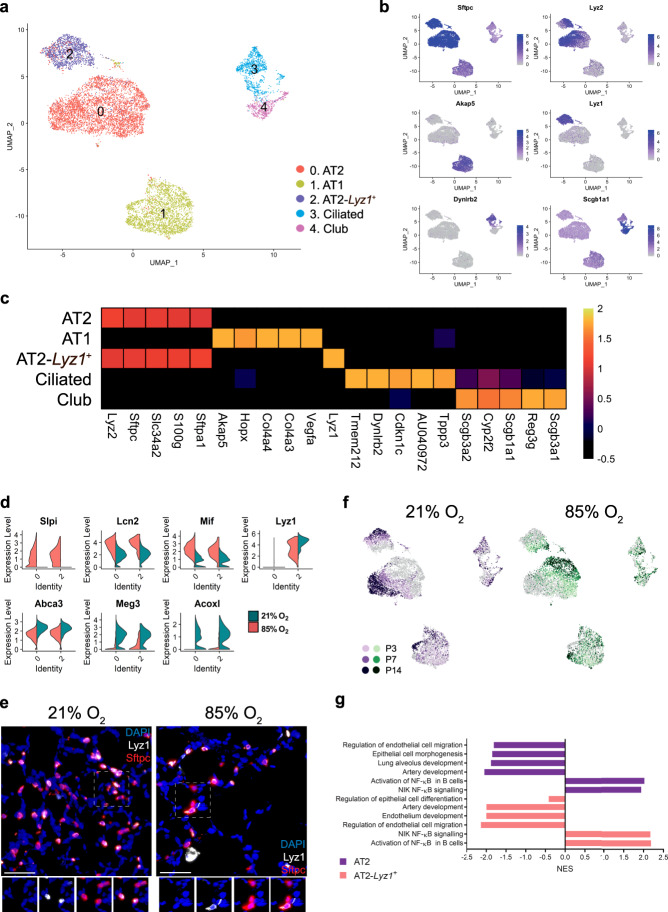
Cellular composition of epithelial cells during normal and hyperoxia-impaired late murine lung development. **a** A total of five clusters of epithelial cells were identified in developing lungs. Cell populations are colored as indicated by the legend. **b** UMAP plots showing expression of principal identifiers of different epithelial cell types. The intensity of expression is indicated by purple coloring. **c** Heatmap of top five most differentially expressed genes across epithelial clusters. The intensity of expression is indicated as specified by the color legend. **d** Violin plots depicting changes in gene expression of some normoxia (21% O_2_, green) and hyperoxia-specific (85% O_2_, red) genes in the two AT2 clusters at P14. **e** Representative images from fluorescent RNA in situ hybridization for AT2-*Lyz1*^+^ marker *Lyz1* (white) and pan-AT2 marker *Sftpc* (red) in developing mouse lungs. Magnification: 40×. Scale bar = 40 µm. Three 14-days old animals/group were analyzed. **f** UMAP plots depicting cell identity in regard to developmental time points in normally (purple) and aberrantly (green) developing lung epithelium. The intensity of expression is indicated as specified by the color legend. **g** Selected hyperoxia-impacted signaling pathways in AT2 (purple) and AT2-*Lyz1*^+^ (pink) clusters, as identified by gene set enrichment analysis (GSEA). All terms are significantly enriched (adjusted *p* value < 0.05) and normalized enrichment scores (NES) are shown. NES values were computed by gene set enrichment analysis on fold change-ranked genes. Expression values in Heatmap and violin plots represent *Z*-score-transformed log(TP10k + 1) values. Expression levels in UMAP plots are presented as log(TP10k + 1) values. Log(TP10k + 1) corresponds to log-transformed UMIs per 10k.

We identified five clusters of epithelial cells with distinct expression profiles (Fig. 3a–c; Supplementary Fig. 4a; Supplementary Data 3).

Two bronchial epithelial clusters—club cells and ciliated cells—were identified. The frequency of club cells in normally developing lungs remained constant between P3 and P7, but decreased slightly by P14. This dynamic was absent in club cells population in hyperoxic lungs (Supplementary Fig. 4b, Supplementary Data 2).

Within the alveolar epithelium, we identified one alveolar epithelial type 1 cell (AT1) cluster and two distinct AT2 clusters (Fig. 3a). The AT1 cluster was associated with expression of *Hopx, Akap5, and Vegfa* (Fig. 3b, c). The proportion of AT1 cells (cluster 1) decreased during normal development. This decline was not observed between P7 and P14 in hyperoxia-exposed lungs (Supplementary Figs. 2a and 4b).

The two AT2 cell clusters had largely similar transcriptional profiles in developing lungs, with *Lyz1* serving as the single most distinctive identifier of the secondary AT2 population (AT2-*Lyz1*^+^; Fig. 3b–d; Supplementary Fig. 4a and c; Supplementary Data 3).

While decreased lysozyme content in airway secretions was associated with BPD^13^, unlike in humans, lysozyme orthologs in mice are encoded by two genes (*Lyz1* and *Lyz2*). The AT2-*Lyz1*^+^ population may be mouse-specific. Both clusters showed shifts in gene expression in hyperoxia-exposed lungs at all investigated time points (Fig. 3d and f; Supplementary Fig. 4a; Supplementary Data 4). Among the hyperoxia-specific genes were multiple factors associated with BPD and epithelial damage, including *Slpi*—a protease inhibitor protecting from epithelial damage—and the innate immune response regulator *Mif*^14^ (Fig. 3d). MIF was previously shown to promote production of IL6 and IL1β, and has been implicated in the arrest of lung development and angiogenesis^15,16^. *Lcn-2* expression—a gene associated with BPD^17^—was also higher in the aberrant AT2 cells. Among the top downregulated genes by hyperoxia were *Meg3* and *Abca3* (Fig. 3d). *Meg3* is a known regulator of epithelial cell differentiation, while *Abca3* is a crucial factor in surfactant and lamellar bodies’ metabolism^18,19^. Mutations of *Abca3* have also been associated with interstitial lung disease and respiratory distress^20^. Furthermore, gene set enrichment analysis (GSEA) of AT2 populations suggested that pathways associated with epithelial, endothelial, and lung alveolus developments were downregulated following hyperoxia exposure (Fig. 3g; Supplementary Data 5).

Finally, we sought to identify a subpopulation of *Axin2*^*+*^ AT2 progenitors, postulated to play a role in alveolar regeneration^21^. We identified a small number of epithelial cells expressing *Axin2*, mostly located to AT1 and AT2 clusters. *Axin2*^*+*^ cells were not confined to a separate cluster, but were primarily present within AT1 and AT2 clusters (Supplementary Fig. 4d).

Cell communication inference suggested that AT2 and AT2-*Lyz1*^+^ are very interactive with other cell types during the response to hyperoxia. This analysis highlighted multiple hyperoxia-related effects in both AT2 clusters, possibly mediated by signals from stromal, endothelial and immune cells, including *Vegfa* and *Tnf* signaling (Fig. 2e; Supplementary Fig. 4e, f). Hyperoxia upregulated *Tnf* expression in interstitial macrophages, which was associated with expression of *Tnf* receptors *Nrp1* and *Dag1* in AT2 cells (Fig. 2e; Supplementary Fig. 4e, f). *Tnf* is a potent pro-inflammatory cytokine increased in both BPD patients and in animal models of BPD^22,23^. *Nrp1* is critical for normal branching morphogenesis^24^, while *Dag1* plays an important role in airway epithelial wound repair^25^. In addition, the analysis revealed a potential regulation of AT2 cells by *Angpt2*, which was upregulated by hyperoxia in two capillary clusters (Cap, Cap-a) (Fig. 2e; Supplementary Fig. 4e). *Angpt2* is a ligand of the cell surface receptor ITGB1, critical in epithelia stratification during lung branching morphogenesis^26^.

### Hyperoxia induces dramatic shifts in developing murine lung stromal Col13a1^+^ fibroblasts, myofibroblast, and pericyte populations

**Fig. 4:**
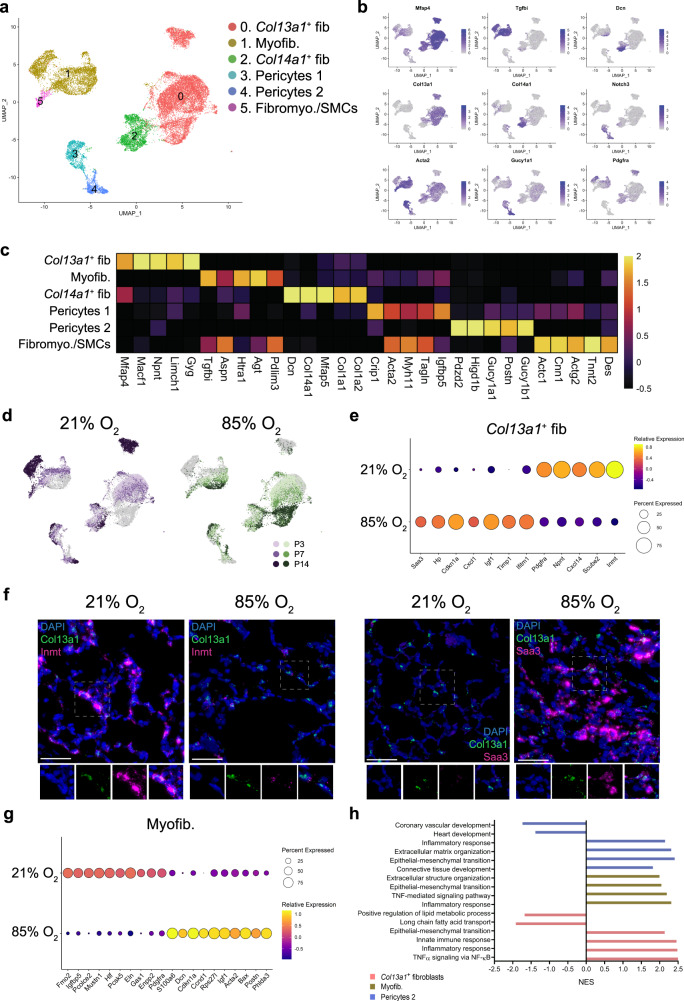
Cellular composition of stromal cells during normal and hyperoxia-impaired late lung development. **a** A total of six clusters of stromal cells were identified in developing lungs. Cell populations are colored as indicated by the legend. **b** UMAP plots showing expression of principal identifiers of different stromal cell types. The intensity of expression is indicated by purple coloring. **c** Heatmap of top five most differentially expressed genes across stromal clusters. The intensity of expression is indicated as specified by the color legend. **d** UMAP plots depicting cell identity in regard to developmental time points in normally (purple) and aberrantly (green) developing lung endothelium. The intensity of expression is indicated as specified by the color legend. **e** Dotplot depicting expression of oxygen-specific markers in *Col13a1*^+^ fibroblasts at P14. The intensity of expression is indicated by the color legend. Size of the cell population expressing the gene of interest is indicated by the size of the circle as specified by the legend. **f** Fluorescent RNA in situ hybridization showing co-expression of *Inmt* (pink) and *Saa3* (pink) with *Col13a1* (green) in normal and aberrant mouse lungs. Magnification: 40×. Scale bar = 40 µm. Two 14-days old animals/group were analyzed. **g** Dotplot depicting expression of oxygen-specific markers in Myofib. cluster at P14. The intensity of expression is indicated by the color legend. Size of the cell population expressing the gene of interest is indicated by the size of the circle as specified by the legend. **h** Selected hyperoxia-impacted signaling pathways in *Col13a1*^+^ fib (pink), Myofib. (yellow), and Pericytes 2 (blue) clusters as identified by gene set enrichment analysis (GSEA). All terms are significantly enriched (adjusted *p* value < 0.05) and normalized enrichment scores (NES) are shown. NES values were computed by gene set enrichment analysis on fold change-ranked genes. Expression values in Heatmap and violin plots represent Z-score-transformed log(TP10k + 1) values. Expression levels in UMAP plots and Dotplots are presented as log(TP10k + 1) values. Log(TP10k + 1) corresponds to log-transformed UMIs per 10k.

Within the stroma, we identified six populations, including pericytes, fibroblasts, and myofibroblasts (Fig. 2a; Fig. 4a–c; Supplementary Data 6).

We identified two clusters of pericytes, distinguished by expression of *Acta2* (Pericytes 1) and *Gucy1a1* (Pericytes 2) (Fig. 4a–c). All pericytes expressed pericyte-specific markers and were negative for the fibroblast marker *Pdgfra*^1,27,28^ (Fig. 4b, c). Both populations increased during development, together representing ~12.5% of stromal cells by P14. While Pericytes 1 were not impacted by hyperoxia, the size of Pericytes 2 cluster was significantly decreased by P14 (Supplementary Figs. 2a and  5a, b).

Two distinct fibroblast clusters were identified (Fig. 4a–c). As proposed previously^29^, we categorized fibroblasts based on the expression of *Col13a1* and *Col14a1* (Fig. 2a, b).

The population of *Col13a1*^+^ fibroblasts expressed additional *Col13a1*^+^ fibroblast markers^29^ and multiple pan-fibroblast markers, as well as the lipofibroblast marker *Plin2* (Supplementary Fig. 5c). The *Col13a1*^+^ fibroblasts cluster represented ∼60% of all stromal cells at P3 in both healthy and diseased lungs (Supplementary Fig. 2a; Supplementary Fig. 5a; Supplementary Data 2). While the size of the population rapidly decreased in healthy lungs, this decrease was largely absent in hyperoxia-exposed lungs. By P14, hyperoxia exposure induced large shifts in the expression profiles of *Col13a1*^+^ fibroblasts (Fig. 4d; Supplementary Fig. 5b; Supplementary Data 7). The top marker for normoxic and hyperoxic *Col13a1*^+^ fibroblasts were *Inmt* and *Saa3*, respectively (Fig. 4e; Supplementary Fig. 5d). To confirm this finding, we further analyzed the expression pattern of *Inmt*/*INMT* and *Saa3*/*SAA3* RNA in *Col13a1*^+^/*COL13A1*^*+*^ fibroblasts in mouse and human BPD lung tissues, respectively (Fig. 4f and Supplementary Fig. 5e, respectively). *Saa3* was increased in a lamb preterm lung injury model^30^ and regulated *Pdgfra*^31^, which is critical for normal alveolar development, and have been reported to be decreased by hyperoxia-exposure^32,33^. In addition, decreased PDGFRA expression was associated with increased risk for male patients to develop BPD^34^. The expression of *Pdgfra* was specifically decreased by hyperoxia in both fibroblast clusters (Fig. 4e; Supplementary Fig. 5f; Supplementary Data 7). This was however not associated with sex genotype within the hyperoxic portion of the *Col14a1*^*+*^ fibroblasts (Supplementary Fig. 5f). While further studies of SAA3-PDGFRA interactions are needed, these data support the hypothesis that PDGFRA (possibly via regulation by SAA3) may play a role in hyperoxia-induced changes in *Col13a1*^+^ fibroblasts.

The *Col14a1*^*+*^ cluster expressed known markers of *Col14a1*^*+*^ fibroblasts, including *Meg3*, *Dcn*, and *Fbln1*^29^. In addition, this population expressed *Col1a1* and *Col1a2*, consistent with the expression signature of interstitial matrix fibroblasts^1,35^ (Supplementary Fig. 5c). Within the cluster, we detected a subpopulation of *Dcn*^+^ cells co-expressing a progenitor marker *Ly6a* (*Sca1*), identifying a potential population of lung resident mesenchymal stromal cells (MSCs; Supplementary Fig. 5g)^36^. The *Ly6a*^+^ population increased significantly between P3 and P14 in both healthy and BPD lungs. Correspondingly, gene expression levels of multiple MSC markers (*Cd44*, *Eng,* and *Lepr*) were increased in *Col14a1*^*+*^ cluster by hyperoxia at P14 (Supplementary Fig. 5g).

Further, we identified one myofibroblast (Myofib.) cluster expressing *Tgfbi* and *Acta2* (Fig. 4b, c). While the size of the myofibroblast population gradually increased during development in the healthy lungs, this increase was less obvious in the diseased lungs (Supplementary Fig. 5a; Supplementary Data 2). This was accompanied by a major transcriptional shift in the myofibroblast population from diseased lungs at P14 (Fig. 4g). GSEA of hyperoxia-induced gene expression further suggested a hyperoxia-induced myofibroblast-mediated activation of extracellular matrix-related and immune pathways (Fig. 4h; Supplementary Data 8).

Finally, we identified an additional small population of fibromyocytes/smooth muscle cells (Fibromyo/SMCs). This cluster expressed a mixture of fibroblast, myofibroblast, and SMC markers, partially corresponding to the fibromyocyte population described by Travaglini et al.^37^ (Fig. 4a–c). The Fibromyo/SMCs cluster displayed limited dynamic during the development and relatively few hyperoxia-induced changes in gene expression (Figs. 2a, 4d; Supplementary Data 7).

Cell communication inference revealed *Col13a1*^+^ fibroblasts, myofibroblasts and Pericytes 2 as potent signal senders and receivers among the stromal clusters in hyperoxia, essentially affecting all other cellular compartments (Fig. 2e). Hyperoxia-induced signals received by stromal cells largely originated from immune cells, particularly alveolar and interstitial macrophages, and from within the stromal compartment itself (Supplementary Fig. 6a). Similar to epithelial cells, this analysis revealed induction of the pro-inflammatory TNF signaling pathway in stromal cells, particularly *Col13a1*^+^ fibroblasts. Additionally, we observed an induction of *Anxa1*, reported to act as a regulator of TNF-induced proliferation and inflammatory responses in lung fibroblasts^38^. This is in agreement with the hyperoxia-mediated induction of multiple immune pathways in *Col13a1*^+^ fibroblasts as suggested by GSEA (Fig. 4h; Supplementary Data 8). This increase in *Anxa1* expression was further associated with increased expression of its receptors *Cd44* and *Vcam1* in *Col13a1*^+^ fibroblasts (Supplementary Fig. 6b; Supplementary Data 7). Increased expression of *Vcam1* was previously reported in the lungs of mechanically ventilated rats^39^ and increased *Cd44* levels were associated with protective effects in hyperoxia-exposed mice^40^. Signals received by Myofib. and Pericytes 2 clusters include multiple extracellular matrix components, including *Mmp9*, *Col4a1*, *Tnc,* and *Adam17* (Supplementary Fig. 6a). MMP9, in our study primarily produced in hyperoxia by neutrophils and Alv. Mϕ clusters were previously postulated to play a role in lung alveolarization and BPD pathology^41^. *Tnc* expression was increased by hyperoxia in all stromal clusters (Supplementary Fig. 6a). *Tnc* contributes to normal alveolarization and development of lung SMCs^42^ and protects from hyperoxia-induced lung injury^43^.

### Hyperoxia induces inflammatory and anti-angiogenic gene expression in murine Car4^+^ capillary endothelial cells

We identified five distinct EC clusters based on their expression profiles. These included arterial, venous, capillary and lymphatic endothelial cells.

**Fig. 5:**
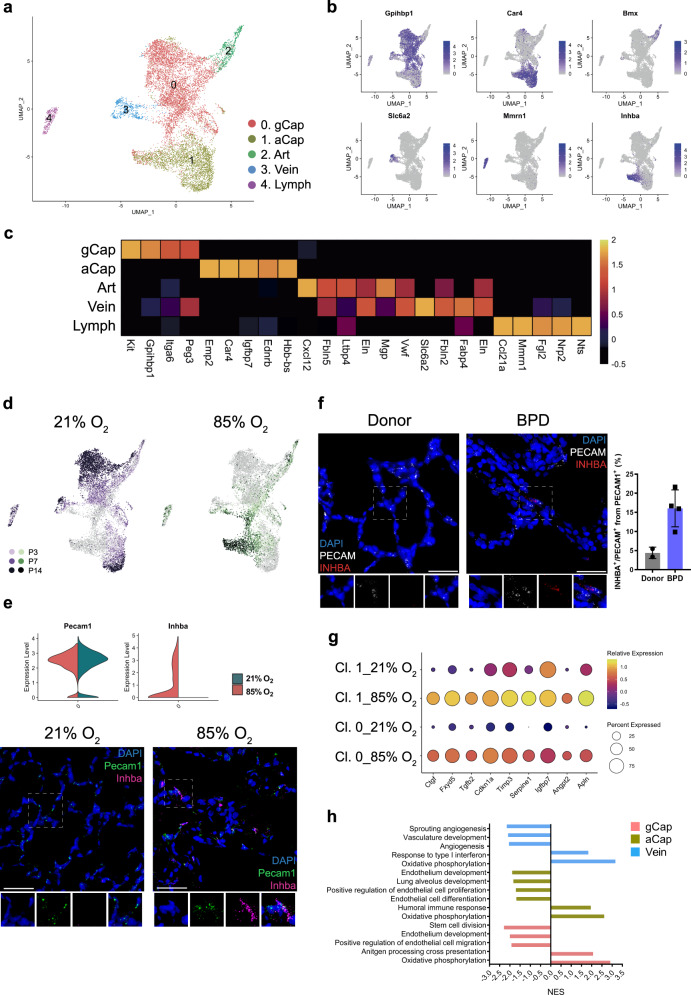
Cellular composition of lung endothelium during normal and hyperoxia-impaired late lung development. **a** A total of five clusters of endothelial cells were identified in developing lungs. Cell populations are colored as indicated by the legend. **b** UMAP plots of principal identifiers of different types of endothelial cells. The intensity of expression is indicated by purple coloring. **c** Heatmap of top 5 most differentially expressed genes across endothelial clusters. The intensity of expression is indicated as specified by the color legend. **d** UMAP plots depicting cell identity in regards to developmental time points in normally (purple) and aberrantly (green) developing lung endothelium. The intensity of expression is indicated as specified by the color legend. Violin plots depicting changes in gene expression of *Pecam* and *Inhba* in normoxia (21% O_2_, green) and hyperoxia-specific (85% O_2_, red) at P14. **e** Representative images from fluorescent RNA in situ hybridization for hyperoxia-specific marker *Inhba* (pink) and pan-endothelial marker *Pecam* (green) in developing mouse lungs. Magnification: 40×. Scale bar = 40 µm. Two 14-days old animals/group were analyzed. **f** Representative images and quantitative analysis of fluorescent RNA in situ hybridization for hyperoxia-specific marker *INHBA* (red) and pan-endothelial marker *PECAM* (white) in BPD patients’ and donors’ lungs. Samples from five BPD patients and two donor lungs were analyzed. For quantitative analysis, cells in 15 randomly chosen fields of view/sample were analyzed. Magnification: 40×. Scale bar = 40 µm. **g** Dot plot depicting hyperoxia-induced changes in the expression of inflammatory, anti-angiogenic and protective genes in capillary cells at P14. The intensity of expression is indicated by the color legend. Size of the cell population expressing the gene of interest is indicated by the size of the circle as specified by the legend. **h** Hyperoxia-impacted signaling pathways in gCap (pink), aCap (yellow), and Vein (blue) clusters, as identified by gene set enrichment analysis (GSEA). All terms are significantly enriched (adjusted *p* value < 0.05) and normalized enrichment scores (NES) are shown. NES values were computed by gene set enrichment analysis on fold change-ranked genes. Expression values in Heatmap represent *Z*-score-transformed log(TP10k + 1) values. Expression levels in UMAP plots and Dotplots are presented as log(TP10k + 1) values. Log(TP10k + 1) corresponds to log-transformed UMIs per 10k.

Capillary cells formed two distinct clusters gCap and aCap^37,44,45^ (Fig. 5a–c; Supplementary Data 9), corresponding to capillary cell phenotypes recently characterized by Gillich et al.^46^. Throughout normal development, the proportion of gCap cells increased significantly (Supplementary Fig. 7a, b; Supplementary Data 2). In contrast to the gCap cluster, the number of aCap cells seemed to decrease with time, indicating the importance of this population in early postnatal development (Supplementary Fig. 7a, b; Supplementary Data 2). Hyperoxia significantly reduced the number of gCap cells (Supplementary Fig. 2a), but increased the number of aCap cells, distinguished by the expression of *Car4* (Fig. 5b–d; Supplementary Fig. 7a, b; Supplementary Data 2). *Car4*^+^ endothelial cells contribute to lung septation^47^ and alveolar revascularization after injury^48^. Our data suggest that hyperoxia particularly affected the gene expression of both gCap and *Car*^*4*+^ aCap cells as assessed by the number of differentially regulated genes (Fig. 5d; Supplementary Fig. 7b; Supplementary Data 10). The top differentially regulated gene in the hyperoxic aCap cells was *Inhba* (Fig. 5e; Supplementary Data 10), a member of the TGFβ superfamily suggested to contribute to the pathology of BPD^49^. To validate this finding, we further showed the expression of *Inhba/INHBA* in *Pecam*/*PECAM* positive endothelial cells in both hyperoxic mouse and human BPD lung tissues and in healthy controls (Fig. 5e, f). Several genes known to be induced by cellular stress or inflammation^50–52^ were upregulated in the hyperoxic aCap cells. We observed increased expression of *Ctgf* and *Fxyd5*, known to contribute to inflammatory lung injury^53,54^ and of *Tgfb2*, shown to be associated with profibrotic responses in the lung^55^ (Fig. 5g). In addition, an anti-angiogenic gene expression profile was observed in hyperoxia, characterized by an increase in expression of *Cdkn1a, Timp3*, *Serpine1*, and *Igfbp7* (Fig. 5g), in accordance with the crucial role of angiogenic growth factors in normal lung development^56^. Consistent with previous reports^50^, the cell cycle inhibitor *Cdkn1a*, which may protect the lung from oxidative stress^57,58^, was overexpressed in hyperoxia (Fig. 5g). Another potentially protective gene that was overexpressed in hyperoxia was *Apln*, which was shown to reduce potential pulmonary inflammation, fibrin deposition, and partially restore alveolarization in rat pups with neonatal hyperoxic lung injury^59^ (Fig. 5g). GSEA of hyperoxia-induced effects in gCap and aCap cells suggested downregulation of several angiogenic, developmental, and stem cell-related pathways in hyperoxia (Fig. 5h; Supplementary Data 11).

Cell communication inference suggested aCap to be an interactive cell type in both sending and receiving signals to and from other cell types in the lung in hyperoxia (Fig. 2e; Supplementary Fig. 7c, d). Several hyperoxia-associated gene expression changes in the aCap cells were predicted to be driven by ligands largely expressed by immune and stromal cells. We noticed *Bmpr2* upregulation in aCap cells along with significant upregulation of its ligand *Bmp5* in *Col13*^+^ fibroblasts. *Bmpr2* protects endothelial cells from dysfunction and loss of *Bmpr2* leads to pulmonary arterial hypertension^60^. In addition, our analysis predicted activation of inflammatory immune response in aCap by activation of several receptors involved in immune cell adhesion (*Itgb1*, *Itga2*, *Icam1* and *2*, *Esam*) with simultaneous upregulation of their ligands in stromal cells such as *Col13*^+^ fibroblasts, myofibroblasts, Pericyte 1 (*Tnc*) and immune cells such as Alv Mϕ, Neut 1, Int Mϕ, γδ T cells, and NK cells (*Itgam* and *Selplg*) (Supplementary Fig. 7c, d).

In contrast to capillary endothelial cells, hyperoxia caused less changes in the venous and only minor changes in the arterial and lymphatic ECs (Fig. 5a–d; Supplementary Data 10). As described in the literature, the expression of arterial-specific genes formed a continuum, with the arterial gene expression present in the Cap cluster, whereas in the Vein cluster, the expression of arterial-specific genes was absent or considerably downregulated (Supplementary Fig. 7e). Venous cell GSEA suggested hyperoxia-induced upregulation of oxidative pathways and IFNγ related pathways, and downregulation of some angiogenic pathways (Fig. 5h; Supplementary Data 11). In hyperoxia, venous cells expressed several putative receptors involved in inflammatory immune response that were predicted to be activated by ligands produced by many immune and stromal cells (*Itgam*, *Selplg*, *Apoe*, *Itga4*, *Anxa1*, *Vcam1*) (Supplementary Fig. 7c, d). In contrast, no significant hyperoxia-related interactions were predicted for the lymphatic and arterial cells.

The EC progenitors in the lung are not well understood^61^. gCap cells expressed *Kit*, a previously reported endothelial progenitor marker^62^ (Supplementary Fig. 7f). Hyperoxia significantly reduced expression of *Kit* after P7, a crucial stage of capillary development in the lung (Supplementary Fig. 7f; Supplementary Data 10). Recently, gCap cells were shown to have progenitor properties and contribute to maintenance and repair of capillary endothelium^46^. In contrast to *Kit*, the expression of *Bst1*^63^ and *Procr*^64^, which are suggested to be specifically expressed by EC progenitors, were upregulated in the lungs by hyperoxia, but the cells did not form a unique progenitor cluster (Supplementary Fig. 7g). This is in concordance with recent findings in adult lungs showing that the regenerative potential of endothelial cells was not restricted to a particular subset of cells^48^.

### Hyperoxia exposure activates murine myeloid macrophages and neutrophils

**Fig. 6:**
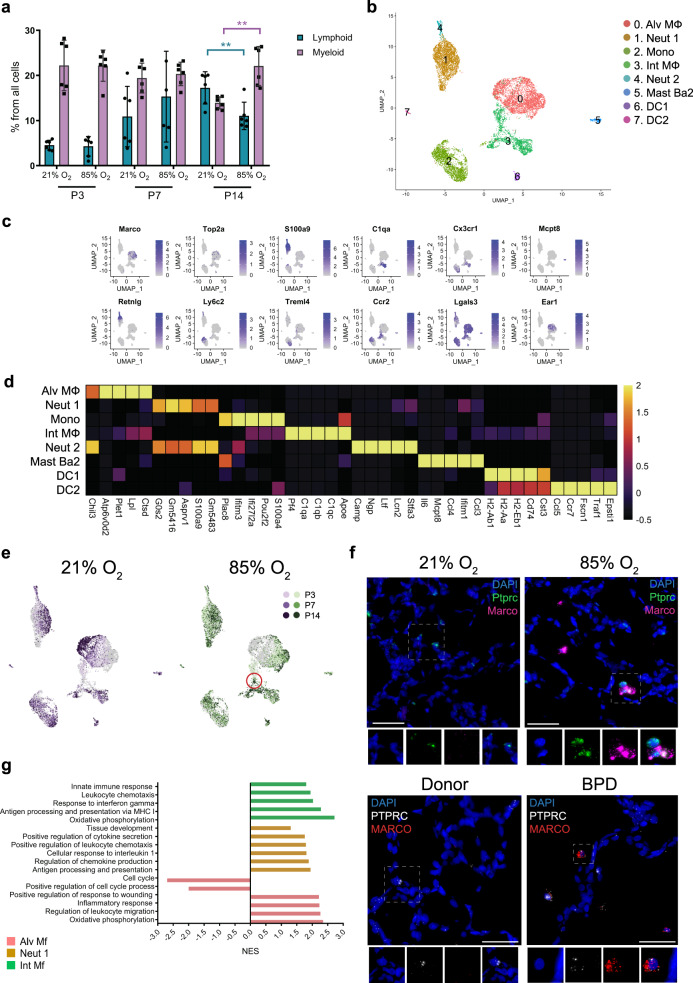
Cellular composition of lung myeloid populations during normal and hyperoxia-impaired late lung development. **a** The relative proportion of myeloid (purple) and lymphoid (teal) cells in developing lungs was significantly impacted by hyperoxia exposure. *n* = 6 animals/group. Data are presented as means ± SD. Statistical analyses were performed with GraphPad Prism 8.0. Significance for each population at each time point was evaluated by unpaired, two-tailed Student’s *t-*test. *P* value = 0.0013 for Myeloid population, and 0.0086 for Lymphoid population. **b** A total of eight clusters of myeloid cells were identified in developing lungs. Cell populations are colored as indicated by the legend. **c** UMAP plots of principal identifiers of different types of myeloid cells. The intensity of expression is indicated by purple coloring. **d** Heatmap of top five most differentially expressed genes across myeloid clusters. The intensity of expression is indicated as specified by the color legend. **e** UMAP plots depicting cell identity of myeloid cells in regard to developmental time points in normally (21% O_2_-exposed, purple) and aberrantly (85% O_2_-exposed, green) developing lung. Each cell is colored by mouse age as indicated by the legend. **f** Fluorescent RNA in situ hybridization showing co-expression of *Marco/MARCO* (pink/red) with *Ptprc/PTPRC* (green/white) positive leukocytes morphologically resembling alveolar macrophages in normal and mouse hyperoxic/human BPD lungs, respectively. Magnification: 40×. Scale bar = 40 µm. Two 14-days old animals/group were analyzed and samples from five BPD patients and two donor lungs were analyzed. **g** Hyperoxia-impacted signaling pathways in Alv Mf (pink), Neut 1 (yellow) and Int Mf (green) clusters as identified by gene set enrichment analysis (GSEA). All terms are significantly enriched (adjusted *p* value < 0.05) and normalized enrichment scores (NES) are shown. NES values were computed by gene set enrichment analysis on fold change-ranked genes. Expression values in Heatmap and violin plots represent *Z*-score-transformed log(TP10k + 1) values. Expression levels in UMAP plots are presented as log(TP10k + 1) values. Log(TP10k + 1) corresponds to log-transformed UMIs per 10k.

Immune cell clusters were identified^1,2,65,66^ (Fig. 2a) and grouped as belonging to either myeloid or lymphoid lineage. During normal lung development, the relative proportion of lymphoid cells from total lung cells increased with time (Fig. 6a). In hyperoxia, the myeloid cells remained the most abundant immune cell type in the lung during development.

We identified eight distinct clusters of myeloid cells, including macrophages, monocytes, neutrophils, dendritic cells (DCs), and basophils (Fig. 6b–d, Supplementary Data 12). Hyperoxia dramatically changed the cell distribution, particularly in the macrophage populations (Fig. 6e; Supplementary Fig. 8a, b).

We identified two distinct macrophage populations, representing alveolar and interstitial macrophages based on the expression of *C1qb*, *Lgals3,* and *Adgre1* gene^37,67^ (Fig. 6b; Supplementary Fig. 8c). In hyperoxia, the number of Alv Mϕ cells decreased, whereas the number of Int Mϕ cells increased (Supplementary Fig. 8b; Supplementary Data 2). Hyperoxia induced the expression of *Inhba* and *Marco* in Alv Mϕ population, whereas the expression of *Ear* was decreased (Supplementary Fig. 8d; Supplementary Data 13). To further validate this finding, we showed that in lung tissue sections from hyperoxic mice and BPD patients, the expression of *Inhba/INHBA* and *Marco/MARCO* was more strongly expressed in *Ptprc/PTPRC* + leukocytes morphologically resembling alveolar macrophages, compared to the healthy control lungs (Fig. 6f and Supplementary Fig. 8e, respectively). GSEA of hyperoxia-induced expression changes in Alv Mϕ cells suggested activation of pathways involved in inflammatory response, leukocyte migration, response to wounding and oxidative phosphorylation (Fig. 6g; Supplementary Data 14). Cell communication inference suggested Alv Mϕ as interactive cell types in sending and receiving signals from other cell types in the lung in hyperoxia (Fig. 2e; Supplementary Fig. 9a, b).

The Int Mϕ population expressed high levels of inflammatory mediators, consistent with data by Gibbings et al.^68^ (Supplementary Data 12). The hyperoxic macrophages clustered together in a particular region of Int Mϕ cluster (Fig. 6e). GSEA of hyperoxia effects in Int Mϕ cells suggested upregulation of pathways involved in oxidative phosphorylation, innate immune response, chemotaxis, and antigen presentation via MHC class I (Fig. 6g; Supplementary Data 14). Of all the lung cell types, cell communication inference suggested Int Mϕ, along with *Col13a*^+^ fibroblasts to be strong mediators of paracrine signaling, releasing factors putatively driving gene expression changes across many cell types (Fig. 2e; Supplementary Fig. 9a, b).

Interestingly, we observed upregulation of *Ccr2* and *Csf1r* in Int Mϕ population after hyperoxia exposure. Furthermore, the expression of their ligands, *Ccl2* and *Csf1*, was upregulated in the Neut and Alv Mϕ, respectively (Supplementary Fig. 9c). These observations are consistent with the study by Kalymbetova et al., where *Ccr2*^−/−^ and CSF1R-depleted mice developed milder structural changes in hyperoxia-exposed lungs^69^.

The concept of M1/M2 polarization of macrophages has been linked to normal development, as well as to several lung pathologies, including BPD^2,70^. Consistent with previous studies, our results suggested that hyperoxia slightly enhanced the M1 signature of both Int Mϕ and Alv Mϕ populations and decreased the M2 signature of AlvMϕ population (Supplementary Fig. 9d). In hyperoxia, several receptors for both M1 (*Ifngr1*, *Ifngr2*, *Adrb2*, *Tnfrsf1b*) and M2 (*Il4ra*, *Il13ra*), as well as receptors associated with both M1- and M2- pathways (*Plaur*, *Cd44*)^71^, were predicted to activate Alv Mϕ cells by ligands expressed from many types of immune, stromal cells, and Cap-a cells according to cell communication predictions (Fig. 2e; Supplementary Fig. 9a, b).

Monocytes are commonly identified as functionally distinct groups of classical or non-classical monocytes. In our study, we found a single monocyte cluster, part of which was positive for classical, and part for non-classical monocyte markers (Fig. 6b; Supplementary Fig. 9a). The number of classical monocytes defined by the expression of *Ly6c2 and Ccr2* increased after hyperoxia exposure, whereas the number of non-classical monocytes defined by the expression of *Fcgr4* was reduced (Supplementary Fig. 9e). These results suggest that, in addition to the hyperoxia-induced upregulation of the *Ccl2*-*Ccr2* axis in macrophages, the number of *Ccr2* expressing classical inflammatory monocytes also increased.

We identified two neutrophil clusters: Neut 1 and 2 (Fig. 6b). Exposure to hyperoxia caused a considerable increase in the size of the Neut 1 cluster and considerable hyperoxia-induced changes in gene expression (Supplementary Fig. 8b; Supplementary Data 2 and 13). GSEA results suggest hyperoxia-induced upregulation of several pathways involved in immune response in these cells (Fig. 6g; Supplementary Data 14). The size and transcriptional profile of the Neut 2 cluster showed fewer changes in hyperoxia compared with Neut 1 (Supplementary Data 13 and 14). Cell communication inference suggested Neut 1 cells as one of the most active cell types in sending signals to other cell types in the hyperoxic lung (Fig. 2e; Supplementary Fig. 9a, b).

### Hyperoxia activates murine lymphoid NK, CD8^+^, and CD4^+^ T cells

**Fig. 7:**
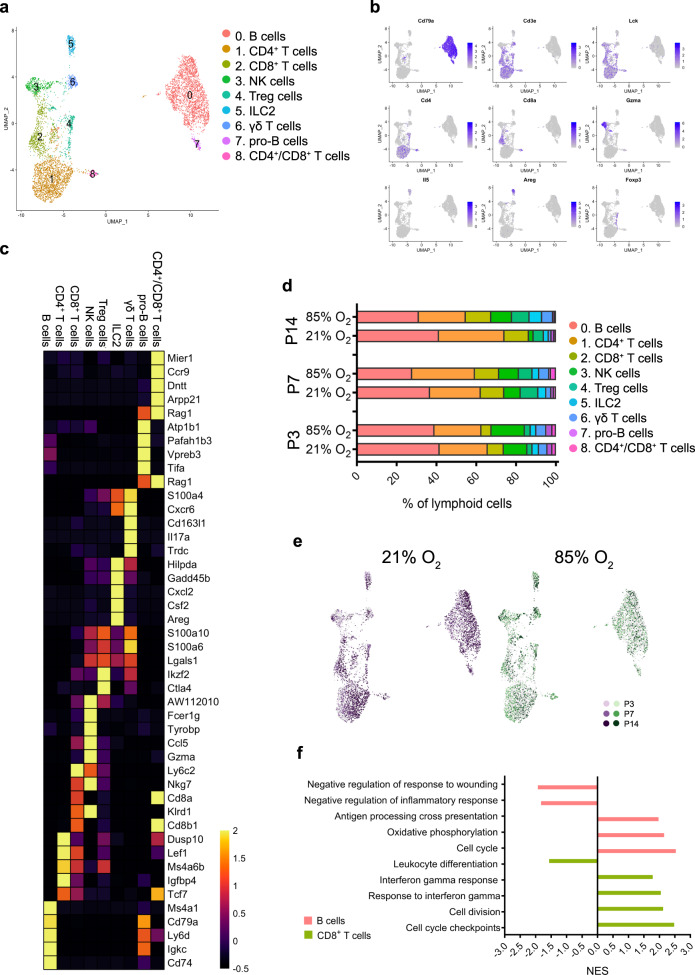
Cellular composition of lung lymphoid populations during normal and hyperoxia-impaired late lung development. **a** A total of nine clusters of lymphoid cells were identified in developing lungs. Cell populations are colored as indicated by the legend. **b** UMAP plots of principal identifiers of identified types of lymphoid cells The intensity of expression is indicated by purple coloring. **c** Heatmap of top five most differentially expressed genes across the lymphoid clusters. The intensity of expression is indicated as specified by the color legend. **d** Relative contribution of individual lymphoid clusters changed significantly during development and exposure to hyperoxia. *n* = 6 animals/group. Cell populations are colored as indicated by the legend. **e** UMAP plots depicting cell identity in regard to developmental time points in (21% O_2_-exposed, purple) normally and aberrantly (85% O_2_-exposed, green) developing lymphoid populations. Each cell is colored by mouse age as indicated by the legend. **f** Hyperoxia-impacted signaling pathways in B cells (pink) and CD8 + T cells (bright green) clusters as identified by gene set enrichment analysis (GSEA). All terms are significantly enriched (adjusted *p* value < 0.05) and normalized enrichment scores (NES) are shown. NES values were computed by gene set enrichment analysis on fold change-ranked genes. Expression values in Heatmap represent *Z*-score-transformed log(TP10k + 1) values. Expression levels in UMAP plots are presented as log(TP10k + 1) values. Log(TP10k + 1) corresponds to log-transformed UMIs per 10k.

Based on known cell markers, we identified nine lymphoid cell clusters including B cells, T cells, NK cells and ILC2 (Fig. 7a–c; Supplementary Data 15). Hyperoxia exposure considerably decreased the proportion of B-cell and CD4^+^ T-cell populations in the lung at P14, whereas the number of NK cells increased significantly (Fig. 7d, e). Differential gene expression and GSEA suggested that the largest changes in expression occurred in the B cell and CD8^+^ T-cell populations and the altered pathways were involved in inflammatory response, response to INFγ, cell cycle, and oxidative phosphorylation (Fig. 7e, f; Supplementary Data 16 and 17). NK cells, CD8^+^ T cells, and Treg cells were predicted to be interactive lymphoid cell types, sending signals to other cell types in the lung driving changes associated with hyperoxia (Fig. 2e). Our results suggest that hyperoxia altered the normal development of adaptive lung immunity due to the hyperoxia-induced activation of the innate immune system, particularly causing a reduction in the number of B-cells and CD4^+^ T cells, resulting in the myeloid cells remaining the major immune cell population of the lung at P14.

### Hyperoxia alters the expression profile of murine mesothelial cells

**Fig. 8:**
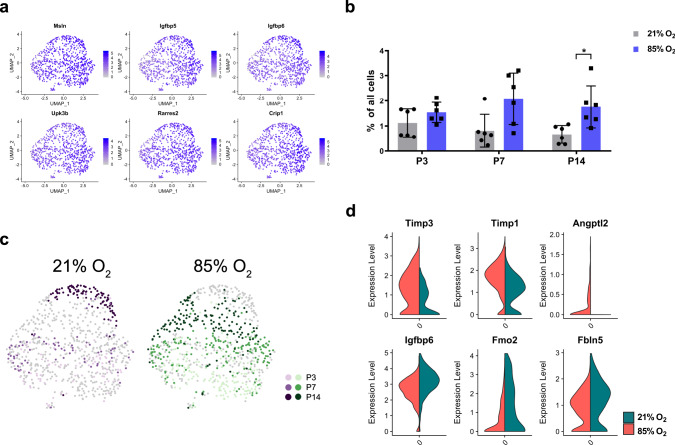
Cellular composition of lung mesothelium during normal and hyperoxia-impaired late lung development. **a** UMAP plots of principal identifiers of mesothelial cells in developing lung. Intensity of expression is indicated by purple coloring. **b** Percentual proportion of mesothelial cells in normally (21% O_2_-exposed, gray) and aberrantly (85% O_2_-exposed, purple) developing lungs at P3, P7 and P14. *n* = 6 animals/group. Significance was evaluated by multiple unpaired multiple Student’s *t-*test with Holm–Sidak correction. Data are presented as means ± SD. *P* value = 0.0426. **c** UMAP plots depicting cell identity in regard to developmental time points in (21% O_2_-exposed, purple) normally and aberrantly (85% O_2_-exposed, green) developing lung mesothelium. Each cell is colored by mouse age as indicated by the legend. **d** Exposure to 85% O_2_ altered gene expression in developing lung mesothelium. 21% O_2_-exposed: green, 85% O_2_-exposed: red. Expression values in violin plots represent *Z*-score-transformed log(TP10k + 1) values. Expression levels in UMAP plots are presented as log(TP10k + 1) values. Log(TP10k + 1) corresponds to log-transformed UMIs per 10k.

A single mesothelial cluster (Fig. 2a, b) was identified based on the expression of *Msln* and other known markers^35^ (Fig. 8a, Supplementary Data 18). Although mesothelial cells represented only 1.20% of all analyzed cells, a clear pattern of gradual decrease was observed in healthy lungs between P3 and P14, which was absent in hyperoxic lungs (Fig. 8b). The developmental arrest induced by hyperoxia was associated with multiple changes in gene expression and signaling pathways (Fig. 8c, d; Supplementary Data 19 and 20). We observed an increase in the pro-angiogenic factor *Angptl2*, accompanied with a decrease in anti-angiogenic *Igfbp6* (Fig. 8d). Among the most de-regulated expression profile were also several components of the extracellular matrix: *Timp1*, *Timp3,* and *Fbln5* (Fig. 8d). FBLN5 is critical for normal alveolar development since Fbln5^−/−^ mice exhibited arrest in alveolarization^41^. Aberrant *Timp1* expression was reported in ventilated preterm human lungs and murine BPD models^41^, while increased *Timp3* expression was associated with BPD severity^72^.

## Discussion

The heterogeneity and origin of lung cell lineages in the context of lung development have been the focus of significant research efforts during the last decades. Here we provide an extensive profiling of cellular composition in normal and impaired late lung development using multiplexed scRNA-seq to assess the expression profile of 66,200 cells. Our study provides insight into the pathogenesis of impaired alveolarization by characterizing several pathological cell populations with distinct molecular expression profiles.

**Fig. 9:**
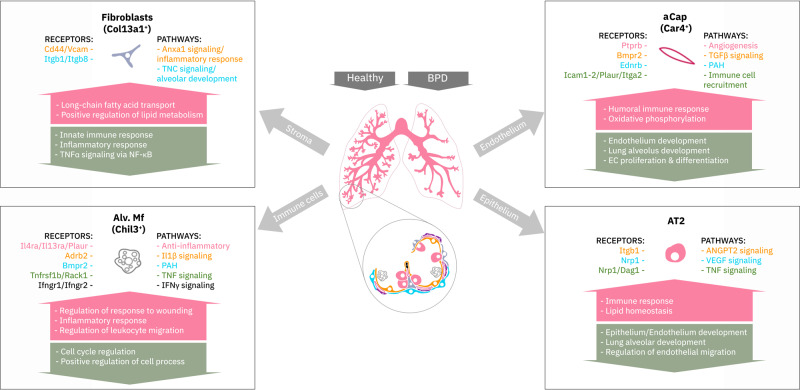
Summary of the cellular crosstalk and pathway analysis in hyperoxia-impaired late murine lung development. Mouse lung cell subtypes with the most significant gene expression changes in hyperoxia mimicking bronchopulmonary dysplasia (BPD). Activated receptors and their involved biological pathways are indicated by the cell communication inference analysis. Biological processes in the arrows are indicated by the gene set enrichment analysis.

We followed the developing lung through three crucial time points of late lung development, across which we identified 5 epithelial, 6 stromal, 5 endothelial, 8 myeloid, 9 lymphoid, and 1 mesothelial cell clusters. Cluster annotations were largely consistent with previously published data^1,2,73^. By assessing tissues at multiple time points, we captured developmental trajectories of these populations during normal and impaired lung development. Hyperoxia exposure altered all cellular compartments, particularly alveolar epithelium, stromal fibroblasts, capillary endothelium, and macrophage populations. Analysis of BPD-associated genes pointed to marked changes in expression in many of the cell populations sensitive to hyperoxia in our data, supporting the importance of these cell populations in the disease process. Pathway analysis and predicted dynamic cellular crosstalk suggested inflammatory signaling as the main driver of hyperoxia-induced changes (Fig. 9).

Importantly, exposure to hyperoxia impaired the composition and expression patterns of all cellular compartments necessary for normal alveolarization. The changes were gradual, appearing from P7 on. Multiple affected transcriptional programs were related to activation of the inflammatory response, suggesting inflammation as one of the main drivers of hyperoxia-induced changes. Previously, fibroblasts (Col13^+^ and Col14^+^), SMCs, macrophages, AT1, and EC lymph cells were predicted to have the strongest network of interactions in lung homeostasis^74^. Our analysis identified hyperoxia-induced interactions between cell compartments in the lung, pointing out lung cell types most actively involved in cellular crosstalk in hyperoxia and specifying the activated receptor pathways. We included selected analyses of interactions induced by hyperoxia, but our dataset also allows further systems level analyses in homeostasis during normal development, not addressed here.

Within epithelial clusters, AT2 and AT2-*Lyz1*^+^ cells were interactive in both sending and receiving signals in the diseased lungs. Signals induced in AT2 clusters originated from stromal, endothelial, and immune compartments.

Within the stroma, Pericytes 2, *Col13a1*^+^ fibroblasts, and myofibroblasts found in diseased lungs showed prominent hyperoxia-induced gene expression pattern changes. *Col13a1*^+^ fibroblasts and Pericytes 2 were further predicted among the most active signal receivers and senders within the developing lung stroma in hyperoxia. Additional signals were received by these cells predominantly from macrophages and epithelial populations. Most dominant pathways included pro-inflammatory and extracellular matrix signaling. Our analysis suggests an important role for *Col13a*^+^ fibroblasts, pericytes and myofibroblasts in hyperoxia exposure.

Particularly striking was a loss of healthy capillary endothelial cells in diseased lungs. Our data support previous studies showing that capillaries are most vulnerable to hyperoxic conditions^61,75,76^. Importantly, hyperoxia seemed to reduce the number of gCap cells, which are putative distal lung vascular progenitor cells^46^. The depletion of these cells may contribute to the lack of repair capability of the injured preterm lung and lead to the occurrence of pulmonary vascular disease^56^ and early onset emphysema in BPD patients^77^, lending some rationale for endothelial-cell-derived therapies^61^. Simultaneously, *Car4*^*+*^ capillary endothelial cells (aCap)^46^, which are important for normal alveolar development and in alveolar revascularization post-injury^47,48^, increased in number. aCap cells displayed a pathological gene expression profile in hyperoxia characterized by upregulation of *Inhba* and many other pro-inflammatory as well as anti-angiogenic markers. aCap were predicted as one of the most active endothelial cell types in hyperoxia-induced cellular cross-talk. The predicted interactions took place with many immune cell types, smooth muscle cells, and epithelial cells. The study by Niethamer et al. described the predicted adult mouse endothelial cell interactions between endothelium and epithelium, using primary epithelial lineages as their epithelial dataset^48^. The authors’ analysis predicted strong interactions between AT1 and *Car4*^*+*^ capillary endothelial cells after influenza exposure^48^, whereas our analysis did not predict any significant hyperoxia-induced interactions between AT1 and aCap cells. However, our data predicted *Angpt2*-mediated signaling between *Car4*^*+*^ aCap cells and AT2 cells.

Our study highlights the importance of activation of the inflammatory response in impaired lung development caused by hyperoxia exposure, which has been previously indicated^78–80^. The majority of hyperoxia-induced signals in the lung were involved in the inflammatory response. In addition to activation of many immune cell types, hyperoxia-induced activation of subpopulations of endothelial, stromal, and epithelial cells, all of which participated in inflammatory signaling. In particular, our data identified innate immune cells such as macrophages and neutrophils as important immune cells in the dynamic cellular cross-talk during hyperoxia. Hyperoxia induced both pro- and anti-inflammatory activation of macrophages, resulting in active signaling from macrophages to other lung cell types. Many of these signaling pathways can be targeted by mesenchymal stromal cell-based therapies, specifically the modulation of macrophages toward an anti-inflammatory phenotype^70^. Also, activation of neutrophils resulted in major inflammatory signaling. In addition to the innate immune cells, the recruitment of lymphoid cells such as NK cells as well as CD8^+^ and CD4^+^ T cells induced inflammatory pathways in many target cell types in hyperoxia. The role of immune cell signaling, such as IL-1β^81^, IFNγ^82^, and TGFβ^83^ has been shown in impaired lung development by several studies. Our data support the importance of these pathways in hyperoxia, as we showed and addressed their activation in specific cell types.

Here we provide data-driven transcriptomic evidence of the most important cell subpopulations and molecular pathways responsible for impaired late lung development caused by hyperoxia exposure. We demonstrate extensive changes in cellular composition caused by hyperoxia, and identify crosstalk between immune, stromal, endothelial, and epithelial cells resulting in impaired lung development. Furthermore, to reflect the relevance of our murine data on the most common chronic lung disease of infancy, BPD, we have validated some of our findings in lung tissues from BPD patients. Limitations of the study include bias from tissue dissociation and bias caused by the low number of cells used for quantification in scRNAseq compared to traditional single-cell methods, such as FACS. These may limit the use of scRNA-seq data for cell quantification. We assessed the magnitude of this bias by stereological cell counting (a gold standard for lung cell quantification) of AT2 cells as an example. However, our analysis is limited to one cell type only. In the future, improved knowledge of markers for cell subpopulations and morphological quantification of these cell populations may provide further insight into the lung dissociation bias. Another limitation was the unequal sex distribution of animals at P3 and P7. However, the largest transcriptional changes were observed at P14, where the sex distribution was equal among study samples. Next, other systems biology approaches such as spatial transcriptomics will allow the construction of a more complete map of events leading to impaired alveolarization. Finally, analyzing the recovery phase after hyperoxia exposure will identify the most severely impaired cell populations unable to recover, which may be responsible for the permanent changes in the lung architecture. In this study, we have described multiple aspects of hyperoxia exposure and identified pathological pathways as putative drug targets of BPD.

## Methods

### Experimental animals

Pregnant C57BL/6 at embryonic day (E)14 or E17 were purchased from Charles Rivers Laboratories, Saint Constant, QC, Canada. Mice were housed by the Animal Care and Veterinary Service of the University of Ottawa in accordance with institutional guidelines. Newborn mouse pups from dams that delivered on the same day, were randomized at day of birth [postnatal day (P) 0] and divided to equal-sized litters of 6 to 8. Following randomization, mice cages were either maintained in room air (normoxia, 21% O_2_) or in normobaric hyperoxia (85% O_2_) from P0 until day of harvest. The hyperoxic environment was maintained in sealed plexiglass chambers with continuous oxygen monitoring (BioSpherix, Redfield, NY). In order to avoid oxygen toxicity and associated confounding factors, nursing dams were rotated between normoxic and hyperoxic group every 48 h. All mice were maintained in 12/12 h light/dark cycle and received food *ad libidum*. All developing mice and their nursing dams were euthanized either at P3, P7 or P14 by 10 μl/g intraperitoneal (i.p.) injection of Pentobarbital Sodium (CDMV, Saint-Hyacinthe, QC, Canada). Animals designated for scRNA-seq and FACS analyses received an additional i.p. injection of 10 mU/g heparin sodium (LEO Pharma INc., Thornhill, ON, Canada). All animal procedures were approved by the Animal Care Committee of the University of Ottawa under animal ethics protocol OHRI-1696.

### Sex genotyping of mice

Determination of sex in mouse pups was performed as described previously^84^. Briefly, genomic DNA was isolated from mice tail cuts and regions of interest were amplified by polymerase chain reaction (PCR) in order to determine expression of male-specific *Sry* gene, as well as *Il3* gene present in mice of both sexes. Forward and reverse primer sequences are listed in Supplementary Table 2. Amplified sequences were visualized by ethidium bromide on 1.5% agarose gel.

### Lung isolation

Pups were euthanized at P3, P7, or P14 by 10 μl/g i.p. injection of pentobarbital sodium (CDMV, Saint-Hyacinthe, QC, Canada). Following euthanasia, mice were tracheotomized and lungs were installation-fixed for 5 min at 20 cm H_2_O hydrostatic pressure with 1.5% (w/v) paraformaldehyde (PFA) (Sigma-Aldrich, Oakville, ON, Canada) and 1.5% (w/v) glutaraldehyde (Sigma-Aldrich, Oakville, ON, Canada) in 150 mM HEPES (Sigma-Aldrich, Oakville, ON, Canada) fixation solution with pH 7.4. After isolation, lungs were kept in the fixation solution for 48 h at 4 °C and collected for embedding in paraffin. Paraffin-embedded tissue blocks were sectioned at 4μm and stained with hematoxylin and eosin (H&E) stain. Tissue dehydration, paraffin embedding, sectioning, and staining were performed by the University of Ottawa Louis Pelletier Histology Core Facility.

### Lung volume measurement

Inflated and fixated lungs were carefully dried with soft tissue in order to remove any droplets. Lung volume was assessed by Archimedes principle (water displacement). The mean value of three measurements/lung was considered.

### Mean linear intercept (MLI) measurement

The MLI was estimated in a blinded fashion using the Quorum Analysis (Quorum Technologies Inc., Guelph, ON, Canada) software. Briefly, MLI quantification was performed in a semi-automated fashion using a 155.34μm line grid moving through sections of interest in defined intervals, where number of intersections between grid line located within alveolar parenchyma and alveolar walls was noted. The average MLI was computed using the formula: ${\mathrm{MLI = }}\left( {{\mathrm{FOVs}} \times \frac{{155.34}}{{\mathrm{I}}}} \right)$, where FOVs = fields of view within which intersections were counted, I = number of intersections and 155.34 = the length of the grid line. A total of at least 200 *FOVs* were assessed in each lung, corresponding to 10 sections analyzed from lungs at P3, 6 sections from lungs at P7 and 4-5 sections from lungs at P14.

### Stereological estimation of number of alveolar type II cells

The number of AT2 cells was determined in the lungs by stereological principles as described previously^85^. Developing pups were euthanized at P14 by 10 μl/g i.p. injection of pentobarbital sodium (CDMV, Saint-Hyacinthe, QC, Canada), tracheotomized and their lungs were installation-fixed for 5 min at 20 cm H_2_O hydrostatic pressure with 4% (w/v) paraformaldehyde (PFA) (Sigma-Aldrich, Oakville, ON, Canada), pH 7.4. Following fixation, lungs were stored in PFA solution for 48 h at 4 °C, embedded *in toto* in 2% agar (Diamed, Mississauga, ON, Canada) and sliced into 2 mm slices. Lung volume was then estimated by the Cavalieri principle exactly as described before^84^. Sliced pieces of each lung were collected for embedding in paraffin. Each paraffin block, containing one pair of lungs from one mouse, was sectioned in agreement with the rules of serial uniform random sampling, where pairs of consecutive, 3 μm thick sections, were collected every 200 sections throughout the block. All sections were stained for the AT2 marker Prosurfactant protein C (ProSPC) and quantified using Stereo Investigator^®^ software (MBF Bioscience, Williston, VT, USA). Essentially, AT2 cells were counted in all consecutive sections at 40× magnification by dissector counting. For every pair of lungs, 0.5% of total surface area was analyzed. The number of cells was calculated using the following formula (1): $N_{({\mathrm{AT}}2)} = \frac{1}{{ssf}} \times \frac{1}{{asf}} \times \frac{1}{2} \times {\sum} {Q_{({\mathrm{AT}}2)}^ - }$_,_ where *ssf* = slide sampling fraction $\left( {\frac{1}{{200}}} \right)$, *asf* = area sampling fraction $\left( {\frac{1}{{200}}} \right)$ and ΣQ^-^_(AT2)_ = number of AT2 as counted in both direction in the dissector setting. The number of AT2 cells was normalized to total surface area of the lung. Surface area was calculated as (2): Sv × N_par_ × V_lung_, where *Sv* = surface density in mm^−1^, N_par_ = parenchymal fraction and V_lung_ = lung volume in mm^3^ as estimated by Cavalieri principle. Parenchymal fraction was assessed as described previously. Surface density was assessed stereologically as described before and calculated using following formula (3): ${\mathrm{Sv}} = \frac{{2 \times I}}{{lp \times {\mathrm{P}}}}$, where Sv = surface density in mm^−1^, I = number of intersections of probe with alveolar surface, *lp* = length of probe/point and P = number of points of the probe falling within parenchymal region of the lung^84^. Tissue dehydration, paraffin embedding, sectioning, and immunohistochemistry were performed by the University of Ottawa Louis Pelletier Histology Core Facility.

### Immunohistochemistry

Briefly, 3 μm thick paraffin sections were deparaffinized in xylene and rehydrated in decreasing ethanol series. Retrieval was accomplished using an ethylenediaminetetraacetic acid (EDTA) buffer (Bond epitope retrieval solution 2, Leica, Concord, ON, Canada), pH9 for 20 min. Sections were stained for proSP-C using an anti-proSP-C antibody (Millipore/Sigma, Entobicoke, ON, Canada) at 1:1500 dilution for 30 min followed by detection with horseradish peroxidase (HRP)-conjugated polymer system (Bond Polymer Refine Detection Kit, Leica, Concord, ON, Canada). All sections were then stained using 3,3′-Diaminobenzidine (DAB) as chromogen, counterstained with Hematoxylin, and mounted.

### Human tissues

Paraffin-embedded lung sections from BPD patients and age-matched donors were kindly provided by the LungMAP Human Tissue Core, Biorepository for Investigation of Neonatal Diseases of Lung-Normal (BRINDL-NL, Dr. Gloria Pryhuber). Written informed consent was obtained from the parents/guardians of the minor participator. Ethical approval was permitted by the University of Rochester, Rochester, NY. Detailed information about all samples is provided in Supplementary Data 21.

### Fluorescent RNA in situ hybridization

RNA in situ hybridization was performed for target detection on fresh 4% PFA-fixed paraffin embedded 3 μm tissue sections using RNAscope Multiplex Fluorescent Reagent Kit Version 2 (Advanced Cell Diagnostics, Newark, CA, USA) according to the manual. Firstly, tissue sections were baked for 1 h at 60 °C, then deparaffinized and treated with hydrogen peroxide for 10 min at room temperature (RT). Target retrieval was performed for 15 min at 98 °C, followed by protease plus treatment for 15 min at 40 °C. All probes were hybridized for 2 h at 40 °C followed by signal amplification and developing of HRP channels was done according to manual. The following RNAscope probes were used in the study: 3-Plex negative control probe dapB (#320871), 3-Plex positive control probe_Mm (#320881), Mm-Inhba-C2 (#455871-C2), Mm-Inmt (#486371), Mm-Marco (#510631), Mm-Pecam1 (#316721), Mm-Saa3 (#446841), Mm-Ptprc-C3 (#318651-C3), Mm-Col13a1-C2 (#837001-C2), Mm-Sftpc-C1 #314101-C1) and Mm-Lyz1-C2 (#415131-C2). For human samples the following probes were used: Hs-INHBA-O1 (#569271), Hs-COL13A1-C2 (#857781-C2), Hs-INMT (#459961), Hs-MARCO (#512231), Hs-PECAM1-No-XMm-O1-C2 (#455931-C2), Hs-PTPRC-C2 (#601991-C2), and Hs-SAA3P (#857771). TSA Plus fluorophores fluorescein Cyanine 3 (1:1500 dilution) and Cyanine 5 (1:3000 dilution) (Perkin Elmer, Waltham, MA, USA) were used for signal detection. Sections were counterstained with DAPI and mounted with ProLong Gold Antifade Mountant (Invitrogen, Carlsbad, CA, USA). Tissue sections were scanned using 3DHISTECH Pannoramic 250 FLASH II digital slide scanner at Genome Biology Unit (Research Programs Unit, Faculty of Medicine, University of Helsinki, Biocenter Finland) using 1 ×40 magnification with extended focus and 7 focus levels. Images were generated using 3DHISTECH Pannoramic 250 FLASH II digital slide scanner at Genome Biology Unit supported by HiLIFE and the Faculty of Medicine, University of Helsinki, and Biocenter Finland.

### Lung isolation and tissue dissociation

Developing pups designated for single-cell RNA sequencing (scRNA-seq) and Fluorescence-activated cell sorting (FACS) analyses were euthanized at P3, P7, or P14 by 10 μl/g i.p. injection of pentobarbital sodium (CDMV, Saint-Hyacinthe, QC, Canada) and received an additional i.p. injection of 10 mU/g (in 10 μl/g volume) heparin sodium (LEO Pharma INc., Thornhill, ON, Canada). Following euthanasia, the chest was opened and the abdominal aorta and vena cava were cut above the liver. The left atrium was perforated and lungs were perfused through the right ventricle with 5 ml of 25 U/ml Heparin Sodium until completely white. Lungs were removed, dissected into individual lobes, and shortly rinsed with Dulbecco’s PBS (DPBS, Lonza, Basel, Switzerland). Dissected lungs were then digested in 5 ml of enzyme mixture at 37 °C by gentleMACS™ Octo Dissociator (Miltenyi Biotech, Bergisch Gladbach, Germany). Following costumed dissociation program was used for the digestion: loop 6× (spin 300 rpm, 10′′; spin −300rpm, 10′′); loop 2× (spin 150 rpm, 5′′; spin −150rpm, 5′′); loop 2× (spin 20 rpm, 5′ 0′′; spin −20rpm, 5′ 0′′); loop 6× (ramp 360 rpm, 15′′; ramp −360rpm, 15′′).

The following customized enzymatic mixture was used for lung digestion: (i) 2500U Collagenase I (Wothington Biochem., Lakewood, NJ, USA), 30U Neutral Protease (Wothington Biochem., Lakewood, NJ, USA), 500U Deoxyribonuclease (DNAse) I (Sigma-Aldrich, Oakville, ON, Canada); (ii) Elastase (Wothington Biochem., Lakewood, NJ, USA), 500U DNAse I; (iii) 2500U Collagenase I, 500U DNase I; iv) Collagenase/Dispase (Sigma-Aldrich, Oakville, ON, Canada), 500U DNAse I. All enzyme mixtures were diluted in 5 ml of DPBS supplemented with Mg^2+^/Ca^2+^ (Thermofischer Scientific, Burlington, ON, Canada). Combination of Collagenase I, Neutral Protease, and DNase (i) resulted in the most equal distribution and was used in all following experiments.

The resulting suspension was filtered through a 100 μm nylon mesh (Thermofisher Scientific, Burlington, ON, Canada) and the enzymatic reaction was terminated by 0.9 mM EDTA. The cell suspension was than centrifuged and the resulting pellet was resuspended in 5 ml DPBS (Lonza, Basel, Switzerland), thoroughly filtered through 40 μm filter (Corning Life Sciences, Tewksbury, MA, USA) and centrifuged again. According to its size, the resulting pellet was resuspended in 500−1000 μl of cold RBC lysis buffer (Thermofischer Scientific, Burlington, ON, Canada) for 3–5 min until white appearance of the suspension was achieved. The cell suspension was than diluted by DPBS (Lonza, Basel, Switzerland) to a total volume of 5 ml, centrifuged and washed twice. Cells were counted using both, the Scepter™ automated cell counter (Millipore-Sigma, Burlington, MA, USA) and a manual hematocyter (Bright-Line™ Hematocyter; Sigma-Aldrich, Oakville, ON, Canada).

### Fluorescent activated cell sorting

The number of cells in single-cell suspension was estimated using a Scepter™ automated cell counter (Millipore-Sigma, Burlington, MA, USA) and a total of 0.5×10^6^ cells/sample were resuspended in 200 μl of PBS in 96-well plate. Cells were incubated in the dark with 2 μl/1 × 10^6^ cells of CD16/32 antibody (Fc block; BD Biosciences, Mississauga, ON, Canada) for 15 min at RT. Following blocking, cells were centrifuged and resulting pellets were resuspended in 1:100 mixture of panel of antibodies: FITC-conjugated CD31 (BD Biosciences, Mississauga, ON, Canada), AF647-conjugated CD45 (Southern Biotech, Birmingham, AL, USA) and Pe/Cy7-conjugated CD326 (EpCAM; Thermofischer Scientific, Burlington, ON, Canada). Cells were incubated with antibodies at RT for 30 min in dark. Following staining, cells were pelleted by centrifugation and washed 3× with FACS buffer (5% (v/v) FBS and 1 mM EDTA in 1×DPBS). All samples were fixed by 4% (w/v) PFA prior to analysis. Flow cytometry was performed using a BD LSR Fortessa (Beckton Dickinson Biosciences, Franklin Lakes, NJ, USA) at the Ottawa Hospital Research Institute (OHRI) core facility. Sample compensation was performed using BD FACSDIVA software and data analysis were performed with FlowJo v10 software (FlowJo LLC, Ashland, OR, USA) (Supplementary Fig. 10).

### Multiplexing individual samples for scRNA-seq

Multiplexing was performed according to the MULTI-seq protocol^8^. Following the preparation of single-cell suspension, cells were counted and a total of 0.5 × 10^6^ cells/sample were resuspended and pelleted in a 96-well plate at 400 × g for 5 min. The resulting pellet was resuspended in 150 μl of 200 nM anchor/200 nM barcode solution (kindly provided by Prof. Zev Gartner from University of California, San Francisco). The lipid-modified DNA oligonucleotide (LMO) anchor and a unique “sample barcode” oligonucleotides were added to each sample in order to be multiplexed, with each sample receiving a different sample barcode (Supplementary Fig. 1e). Samples were then incubated for 10 min at room temperature (RT). After 10 min, samples were supplemented with 200 nM common lipid-modified co-anchor to stabilize the membrane residence of barcodes. Samples were incubated on ice for additional 5 min and pelleted at 400 × *g* for 5 min. Barcode-containing media was then removed, and the resulting cell pellet was washed twice with 1% FBS (Sigma-Aldrich, Oakville, ON, Canada) in 1× DPBS (Lonza, Basel, Switzerland). After the final wash, cells were resuspended in 1× DPBS + 1% FBS, counted and samples were pooled together at 1:1 ratio while maintaining the final concentration of 500-1000 cells/μl. Viability and cell counts were assessed using a manual hematocyter (Bright-Line™ Hematocyter; Sigma-Aldrich, Oakville, ON, Canada), and only samples with viability ≥ 80% were further processed by 10× Chromium.

### scRNA-seq library preparation and sequencing

Single-cell suspensions were processed using the 10x Genomics Single Cell 3′ v3 RNA-seq kit. Gene expression libraries were prepared according to the manufacturer’s protocol. MULTI-seq barcode libraries were retrieved from the samples and libraries were prepared independently, as described previously^8^. Final libraries were sequenced on the NextSeq500 platform (Illumina) to reach an approximate depth of 20,000–25,000 reads/cell.

### Processing of raw sequencing reads

Raw sequencing reads from the gene expression libraries were processed using CellRanger v3.0.2, aligning reads to the mm10 build of the mouse genome. Except for explicitly setting–expect-cells=25000, default parameters were used for all samples. MULTI-seq barcode libraries were simply trimmed to 28 bp using Trimmomatic (v0.36) prior to demultiplexing.

### Demultiplexing expression data with MULTI-seq barcode libraries

Demultiplexing was performed using the deMULTIplex R package (v1.0.2) (https://github.com/chris-mcginnis-ucsf/MULTI-seq). The key concepts for demultiplexing are described in McGinnis et al.^8^. Briefly, the tool considers the barcode sequencing reads and counts the frequency with which each of the sample barcodes appears in each cell. Then, for each barcode, the distribution of counts in cells is assessed and an optimal quantile threshold to deem a cell positive for a given barcode is determined. Cells positive for more than one barcode are classified as doublets and removed. Only cells positive for a single barcode are retained for further analysis (Supplementary Fig. 1b). As each barcode corresponds to a specific sample in the experiment, the sample annotations can then be added to all cells in the data set.

### Data quality control, integration, and clustering

All main processing steps were performed with Seurat v.3.1.5^86^. Quality control was first performed independently on each library to find appropriate filtering thresholds for each. Expression matrices for each sample were loaded into R as Seurat objects, retaining only cells in which more than 200 genes were detected. Poor quality cells with a high percentage (>20%) of UMIs mapped to mitochondrial genes were removed.

We then split each unique sample (based on MULTI-seq sample barcodes) into a separate Seurat object. SCTransform^87^ was used to normalize each sample and select highly variable genes. This was also used to regress out effects of cell cycle and cell stress (percentage of mitochondrial reads) to ensure that downstream clustering would effectively capture cell type patterns rather than cell cycle stage, for example. To further ensure that clustering would not be impacted by batch effects or biological variability associated with mouse age or oxygen conditions, we performed the data integration method implemented by Seurat v3 for SCTransform-normalized data, using the SelectIntegrationFeatures(), PrepSCTIntegration(), FindIntegrationAnchors(), and IntegrateData() functions with default options. PCA was then run on the top 3000 variable genes and the data was then clustered at a low resolution (dims=1:30, Resolution=0.2) with the Louvain algorithm implemented in the FindClusters() function in Seurat.

To identify major cell type subsets (epithelial, endothelial, immune, stromal, mesothelial), we assessed expression of the canonical markers *Epcam, Pecam1, Ptprc, Col1a1*, and *Msln*, respectively. We also assessed additional genes discriminating these clusters by performing a simple Wilcoxon rank-sum test with the FindAllMarkers() function in Seurat. We next isolated each major subset, and reprocessed the subsets using the same normalization and integration approach. We first clustered the data at a higher resolution (subset-specific) and removed rare clusters comprising dozens to a couple hundred of cells expressing canonical markers of contaminant cell types in the subset (e.g., a *Ptprc*^+^ cluster in the epithelial subset), which likely represent rare doublets that were not identified during demultiplexing. With contaminants removed, we re-clustered the integrated data to identify the individual cell types comprising the subset based on previously reported expression signatures.

Cell type labels for each subset were then added to the Seurat object containing all data. While the integration approach prevented clustering and cell-type annotation from being driven by biological and technical effects, these signals are important for data exploration. While retaining integrated cell type labels, we reprocessed data without integration. SCTransform was used to normalize the data, identify the top 3000 variable features, and regress out cell cycle and cell stress. PCA was then performed and UMAP embeddings were generated on the top 40 PCs. This ultimately resulted in UMAP embeddings that show variation associated with experimental conditions with labels resulting underlying cell types.

### Differential expression analysis and gene set enrichment analysis (GSEA)

To identify genes differentially expressed in response to hyperoxia or as a result of mouse age, we used the R package *muscat* (v1.1.6), which was designed specifically for multi-sample, multi-condition scRNA-seq experiments. We used a standard workflow with the tool, generating pseudobulk expression profiles for each sample in each cluster and then testing for differential expression between groups associated with the queried experimental conditions. Significant genes were those with an adjusted *p* value <0.05 and a detection rate of at least 10% in at least one of the conditions tested.

To identify gene sets associated with differentially expressed genes, we used R package *fgsea* (v1.12.0) on the fold-change-ranked list of genes for a given condition. We queried and aggregated a list of gene sets comprising all GO terms, KEGG pathways, Reactome pathways, and the MSigDB Hallmark gene sets. These gene sets were acquired from the Molecular Signatures Database (v6)^88,89^. Significantly enriched gene sets were those with an adjusted *p* value of <0.05 and the normalized enrichment score (NES) was used to assess whether these gene sets were associated with upregulated or downregulated genes in a given condition.

### Cell communication inference

As we were specifically interested in understanding cell communication networks that give rise to hyperoxia-specific effects, we used the R package NicheNet^12^. NicheNet uses information about expression of cognate ligands, receptors, signaling pathways, and genomic targets to infer cell communication patterns that contribute to a specific queried gene set. Differential gene expression in hyperoxia vs normoxia was used for the queried gene set of interest in the NicheNet analysis. To prioritize results, we only performed this analysis to identify signaling that could contribute to the effects in cell types with fairly large responses to hyperoxia (over 200 differentially expressed genes), but included all cell types as potential sources of ligand expression. Background expression of genes was specified with default approach used in NicheNet’s pipeline, i.e. all genes with >10% detection in a given cluster. Cells from both normoxia and hyperoxia conditions were used in the analysis, but in our synthesis of most relevant changes, we prioritized hyperoxia-induced ligands or those from cell types that increase in proportion in hyperoxia samples. For each “receiver” cell type, we selected the top 10 ligands predicted to drive hyperoxia responses based on the Pearson correlation coefficient between the ligand-target regulatory potential score of each ligand and the target indicator vector, as implemented in NicheNet^12^. In each case, we also assessed whether the specific ligands and receptors were upregulated in hyperoxia samples, or whether the cell types expressing the ligands increase in proportion in hyperoxia samples. This information is all included in heatmaps provided in Supplementary Figures. The circos plot in Fig. 2e represents a summary of conditions where the ligands are upregulated in hyperoxia, or the cell type expressing the ligand increases in proportion.

### Statistical analysis

Data are presented as means ± SD. All statistical analyses were performed with GraphPad Prism 8.0. The presence of potential statistical outliers was determined by Grubbs’ test. Differences in case of two-member groups were evaluated either by unpaired Student’s *t*-test, or multiple Student’s *t*-test with correction for multiple comparisons using Holm–Sidak method. *P* values < 0.05 were considered as significant and depicted as following: *P* values < 0.05: *; *P* values < 0.01: **; *P* values < 0.001: ***; *P* values <0.0001: ****.

### Reporting summary

Further information on research design is available in the Nature Research Reporting Summary linked to this article.

## Supplementary information

Supplementary Information

Description of Additional Supplementary Files

Supplementary Movie 1

Supplementary Data 1

Supplementary Data 2

Supplementary Data 3

Supplementary Data 4

Supplementary Data 5

Supplementary Data 6

Supplementary Data 7

Supplementary Data 8

Supplementary Data 9

Supplementary Data 10

Supplementary Data 11

Supplementary Data 12

Supplementary Data 13

Supplementary Data14

Supplementary Data 15

Supplementary Data16

Supplementary Data17

Supplementary Data 18

Supplementary Data 19

Supplementary Data 20

Supplementary Data 21

Reporting Summary

## Data Availability

All scRNA sequencing data, including raw fastq sequencing files, gene expression matrices, and associated cell metadata generated in this study have been deposited in the NCBI’s Gene Expression Omnibus (GEO) database under accession code G4.

## Code availability

Code used for the analysis of scRNA-seq data is available at the public GitHub repository at https://github.com/dpcook/thebaud_lung_BPD.
