# Supplementary material for: Single cell transcriptomic analysis of murine lung development on hyperoxia-induced damage
Source: Nat Commun. 2021 Mar 10;12:1565. doi: 10.1038/s41467-021-21865-2 (PMC7946947; doi:10.1038/s41467-021-21865-2)
Supplement: Supplementary file 1 — Supplementary Information [file 41467_2021_21865_MOESM1_ESM.pdf]

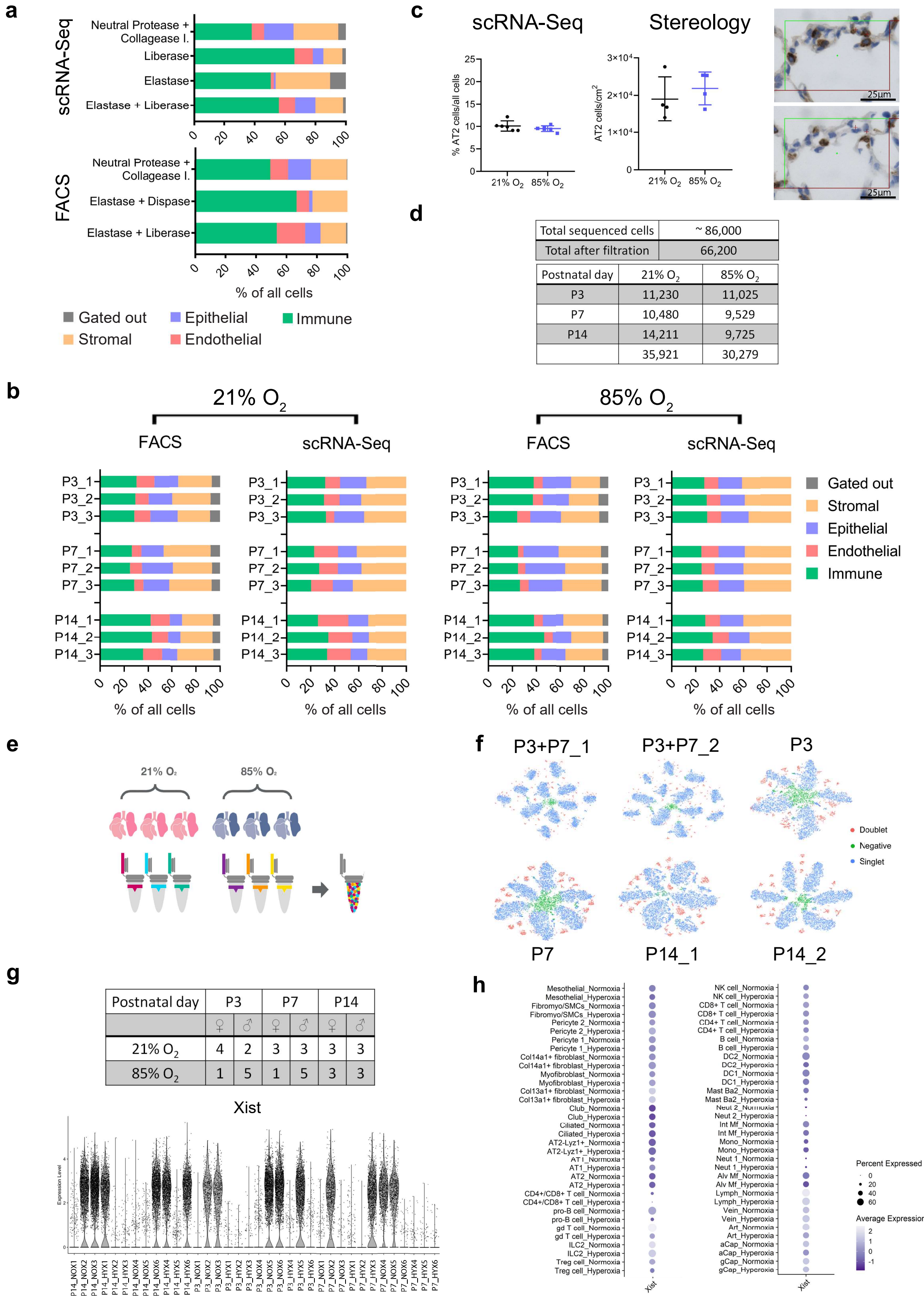

Total sequenced cells	~ 86,000
Total after filtration	66,200

Postnatal day	21% O_2	85% O_2
P3	11,230	11,025
P7	10,480	9,529
P14	14,211	9,725
	35,921	30,279

Postnatal day	P3		P7		P14	
	♀	♂	♀	♂	♀	♂
21% O_2	4	2	3	3	3	3
85% O_2	1	5	1	5	3	3

Supplementary figure 1. Optimization of single-cell isolation protocol.

a) Relative contribution of immune, epithelial, endothelial and stromal cells after digestion with various enzyme buffers as assessed by FACS and scRNA-seq analysis. n = 3 animals/group. Cell populations are colored as indicated by the legend. b) Comparison of the relative contribution of immune, epithelial, endothelial and stromal cells after single-cell isolation between individual lungs isolated at P3, P7 and P14 as assessed by FACS and scRNA-seq analysis. n = 3 animals/group. Cell populations are colored as indicated by the legend. c) Relative proportion of alveolar epithelial type 2 (AT2) cells in normally (black circles) and hyperoxia-impaired (purple squares) developing lungs at P14 assessed by scRNA-seq (n= 6 animals/group) and by stereology (n= 4 animals/group). Representative lung sections illustrate AT2-specific Pro-SPC staining and counting frame used for stereological assessment. Data are presented as means ± SD. Statistical analyses were performed with GraphPad Prism 8.0. The presence of potential statistical outliers was determined by Grubbs' test. Significance was evaluated by unpaired, two-tailed Student's *t*- test. d) Distribution of analyzed cells across individual experimental groups. e) Single cell suspensions from individual lungs were multiplex-labeled and pooled together prior to scRNA-seq in order to eliminate batch variability. Different colours of sample tubes represent samples from different individuals. f) tSNE plots from independent sets of scRNA-seq demonstrating the multiplex bar-labeling of individual samples within each batch. n = 6 or 12/experiment. g) Sex distribution as determined by PCR and expression of *Xist* gene. Expression values represent Z-score-transformed log(TP10k+1) values. h) Dotplot representing the sex distribution in all 34 cell clusters at P14. The intensity of expression is indicated by the color legend. Size of the cell population expressing the gene of interest is indicated by the size of the circle as specified by the legend. Expression levels are presented as log(TP10k+1) values. Log(TP10k+1) corresponds to log-transformed UMIs per 10k.

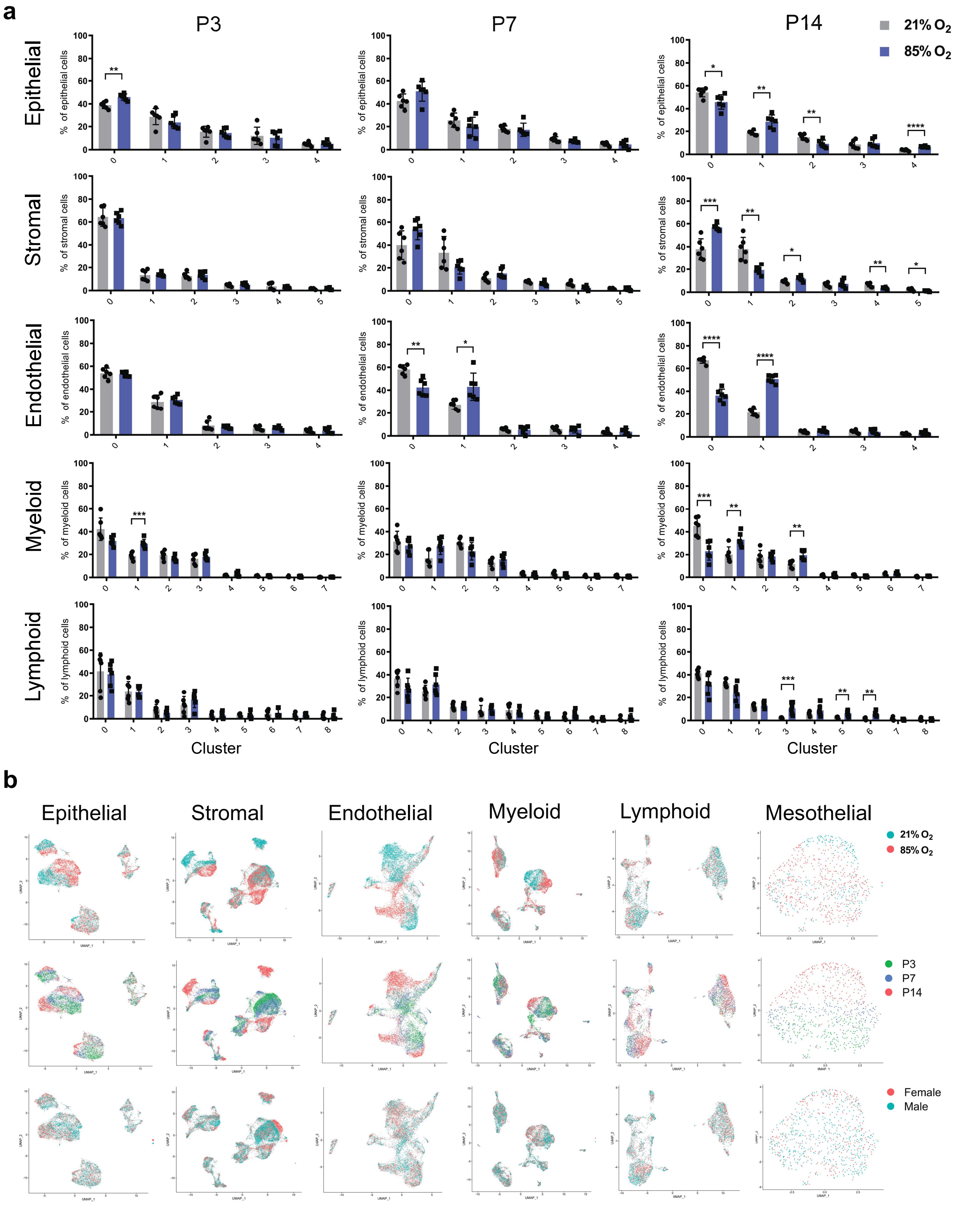

Supplementary figure 2. Cellular composition, and cell distribution in normal and hyperoxia-impaired late lung development.

a) The relative contribution of individual clusters within immune, endothelial, stromal, epithelial and mesothelial cells in normal (grey bars) and aberrantly developing (purple bars) lungs at P3, P7 and P14. Data are presented as means ± SD. Statistical analyses were performed with GraphPad Prism 8.0. The presence of potential statistical outliers was determined by Grubbs' test. Significance was evaluated by multiple unpaired Student's *t*- test with Holm-Sidak correction. *P* values < 0.05: *; *P* values < 0.01: **; *P* values < 0.001: ***; *P* values < 0.0001: ****; n =6 animals/group. b) UMAP plots representing distribution of epithelial, stromal, endothelial, myeloid, lymphoid and mesothelial cells based on oxygen experimental conditions, timepoint and sex. Expression levels are presented as log(TP10k+1) values. Log(TP10k+1) corresponds to log-transformed UMIs per 10k.

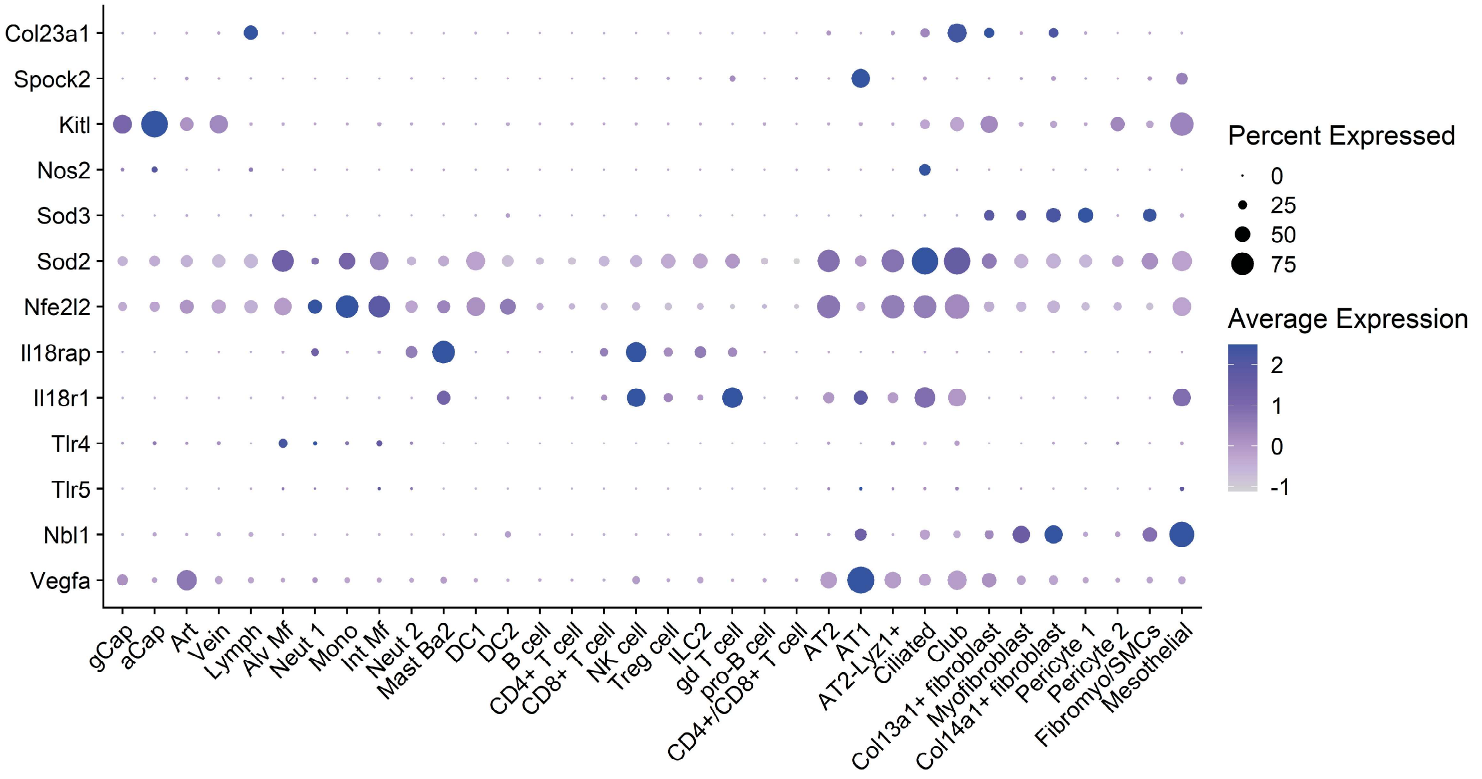

Supplementary figure 3. Expression patterns of genes associated with BPD in normal and hyperoxia-impaired late lung development.

Dotplot displaying expression patterns of genes known to be associated with BPD across all cell populations. The intensity of expression is indicated by the color legend. Size of the cell population expressing the gene of interest is indicated by the size of the circle as specified by the legend. Expression levels in Dotplot are presented as log(TP10k+1) values. Log(TP10k+1) corresponds to log-transformed UMIs per 10k.

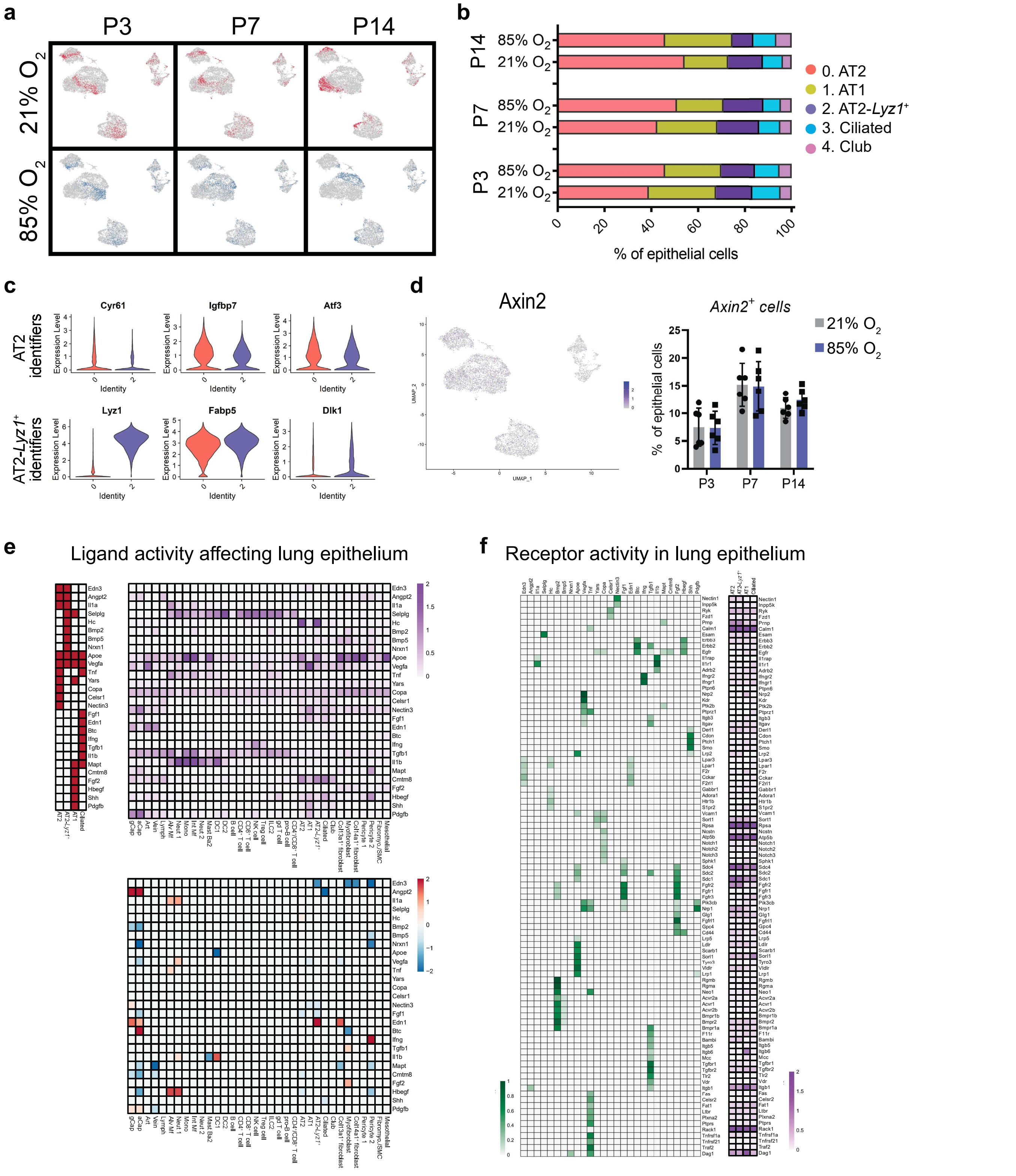

Supplementary figure 4. Cellular composition of normal and hyperoxia-impaired developing lung epithelium. a) UMAP plots representing the temporal changes in gene expression in lung epithelium in normally (red-coloured cells) and aberrantly developing lungs (blue-coloured cells). b) Relative contribution of individual clusters changed significantly during the development and after exposure to hyperoxia. Cell populations are colored as indicated by the legend. n = 6 animals/group c) Violin plots depicting expression patterns of top differentially expressed genes in AT2 (cluster 0, red colour) and AT2-Lyz1^{+} (cluster 2, purple colour) cells. d) UMAP plot of principal identifiers of mesothelial cells in developing lung. The intensity of expression is indicated by purple coloring. Relative contribution of *Axin2*$^{+}$ cells in normally (grey bars), or aberrantly (purple bars) developing lung epithelium at P3, P7 and P14. n =6 animals/group. Significance was evaluated by multiple unpaired Student's *t*-test. Data are presented as means ± SD. e) Heatmaps depicting ligand activity affecting epithelial cells: Grey-red colormap corresponds to whether a ligand was in the top 10 ligands predicted to affect the specific cell type (red colour); Purple heatmaps: Colormap represents average log(TP10k+1) expression values of ligands for each cell type in the hyperoxia samples (depicted in violet); Blue-red colormap represents the log(fold change) expression of ligands in hyperoxia samples (depicted in red/blue). f) Heatmaps of receptor activity showing the putative receptors for each ligand. Green colormap represents the prior interaction potential in NicheNet's model (depicted in green); Purple colormap represents average log(TP10k+1) expression values of receptors for each cell type (depicted in violet). Expression values in violin plots represent Z-score-transformed log(TP10k+1) values. Expression levels in UMAP plots are presented as log(TP10k+1) values. Log(TP10k+1) corresponds to log-transformed UMIs per 10k.

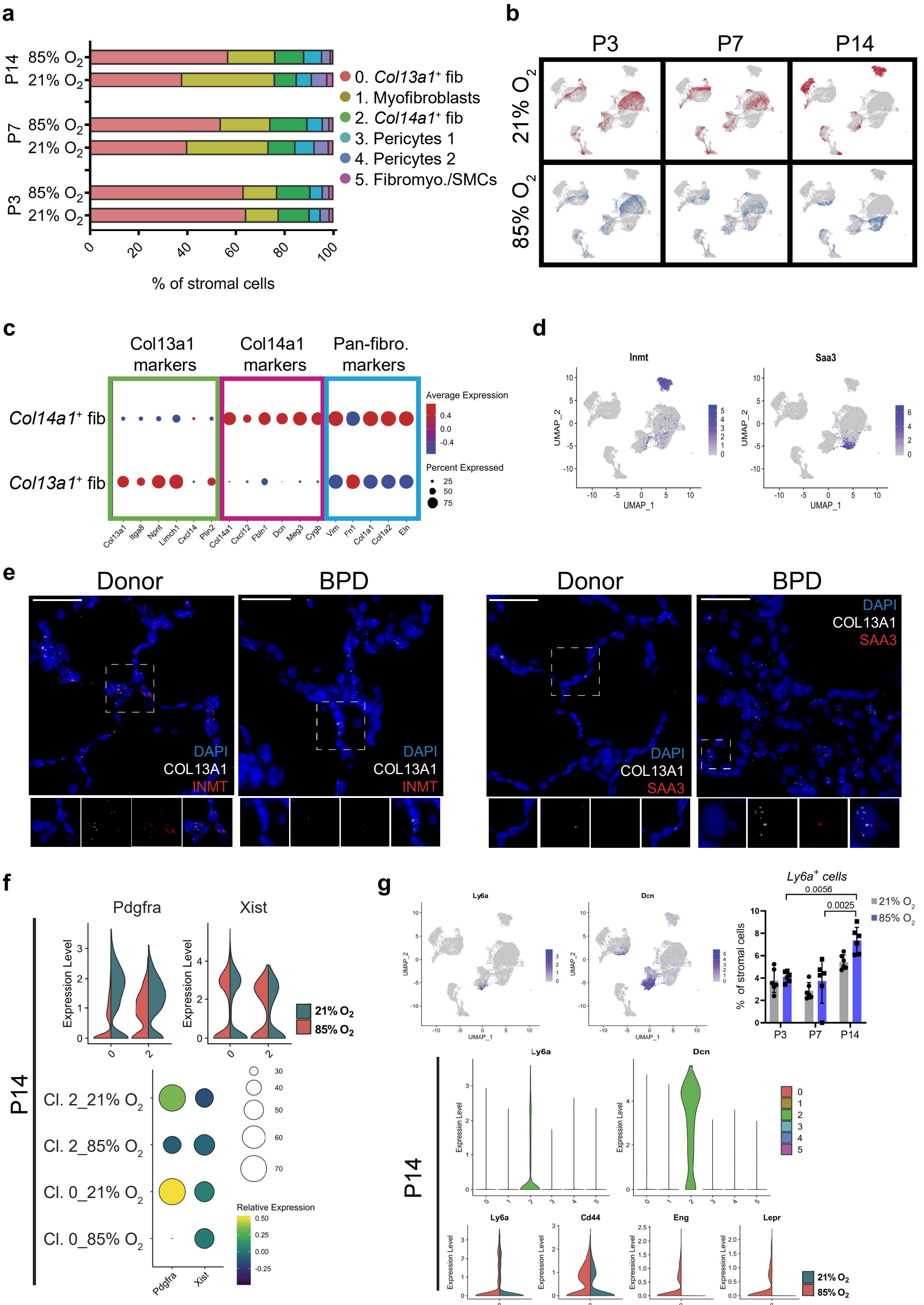

Supplementary figure 5. Cellular composition of normal and hyperoxia-impaired developing lung stroma. a) The relative contribution of individual clusters changed significantly during the development and after exposure to hyperoxia. Cell populations are colored as indicated by the legend. n = 6 animals/group b) UMAP plots representing the temporal changes in gene expression in lung stroma in normal (red-coloured cells) and aberrantly developing lungs (blue-coloured cells). c) Dotplot displaying expression patterns specific to diverse fibroblasts populations. The intensity of expression is indicated by the color legend. Size of the cell population expressing the gene of interest is indicated by the size of the circle as specified by the legend. d) UMAP plots of *Inmt* and *Saa3*. The intensity of expression is indicated by purple coloring. e) Fluorescent RNA in situ hybridization showing co-expression of INMT (red) and SAA3 (red) with COL13A1 (white) in normal and BPD lungs, respectively. Magnification: 40×. Scale bar = 40µm. Samples from five BPD patients and two donor lungs were analyzed. f) Violin plot and Dotplot depicting oxygen-specific expression of Pdgfra in fibroblast clusters at P14. The intensity of expression in Dotplot is indicated by the color legend. Size of the cell population expressing the gene of interest is indicated by the size of the circle as specified by the legend. g) UMAP plots depicting specific localization of *Ly6a*+ cells within *Col14a1*+ fibroblast cluster. The intensity of expression is indicated by purple coloring. The number of *Ly6a*+ cells and expression of stem cell-markers were specifically increased in developing lungs exposed to hyperoxia (purple bars vs. grey bars). Statistical analyses were performed with GraphPad Prism 8.0. The presence of potential statistical outliers was determined by Grubbs' test. n =6 animals/group. Significance was evaluated by 2way ANOVA with Tukey correction. Data are presented as means ± SD. Violin plots depicting specific localization of Ly6a+ cells within Col14a1+ fibroblast cluster (cluster 3, green). Violin plots depicting oxygen-specific expression of mesenchymal stromal cells markers by Col14a1+ fibroblasts. Expression values in violin plots represent Z-score-transformed log(TP10k+1) values. Expression levels in UMAP plots and Dotplot are presented as log(TP10k+1) values. Log(TP10k+1) corresponds to log-transformed UMIs per 10k.

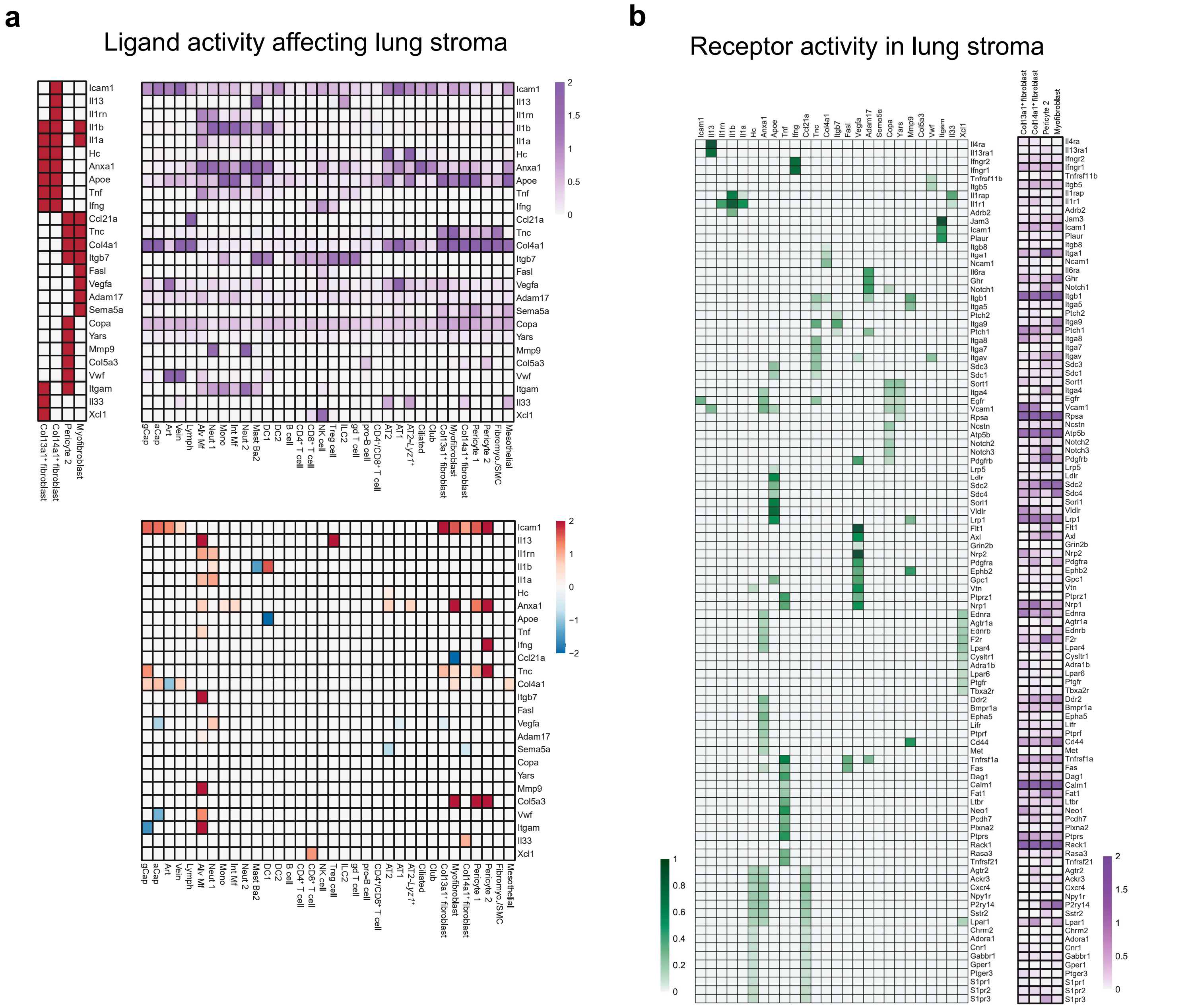

Supplementary figure 6. Cellular communication affecting the developing lung stroma.

a) Heatmaps depicting ligand activity affecting stromal cells: Grey-red colormap corresponds to whether a ligand was in the top 10 ligands predicted to affect the specific cell type (colored red); Purple heatmaps: Colormap represents average log(TP10k+1) expression values of ligands for each cell type in the hyperoxia samples (depicted in violet); Blue-red colormap represents the log(fold change) expression of ligands in hyperoxia samples (depicted in red/blue). b) Heatmaps of receptor activity showing the putative receptors for each ligand. Green colormap represents the prior interaction potential in NicheNet's model (depicted in green); Purple colormap represents average log(TP10k+1) expression values of receptors for each cell type (depicted in violet). Expression values in violin plots represent Z-score-transformed log(TP10k+1) values. Expression levels in UMAP plots and Dotplot are presented as log(TP10k+1) values. Log(TP10k+1) corresponds to log-transformed UMIs per 10k.

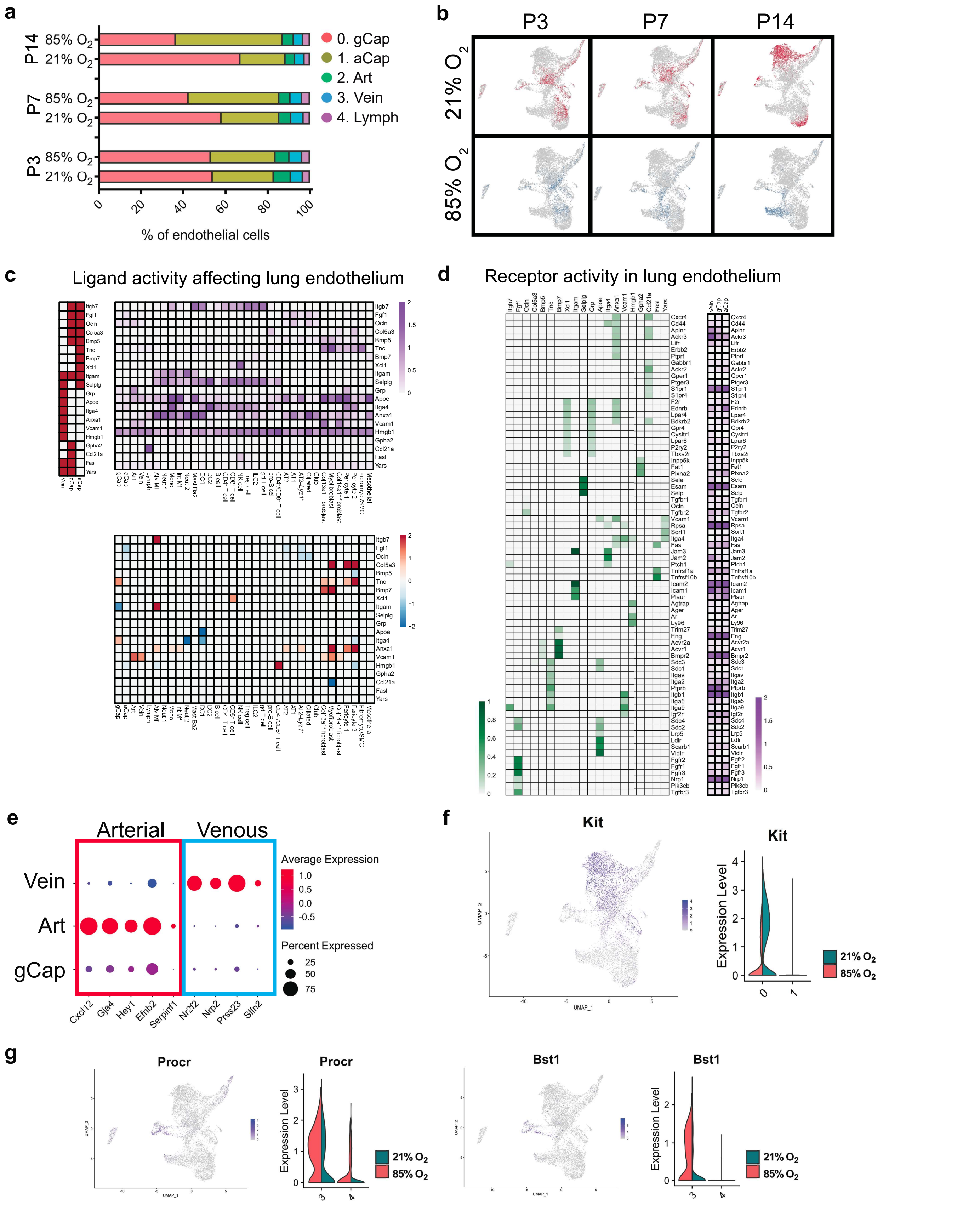

Supplementary figure 7. Cellular composition of normally and hyperoxia-impaired developing lung endothelium.

a) Relative contribution of individual clusters changed significantly during the development and after exposure to hyperoxia. Cell populations are colored as indicated by the legend. n = 6 animals/group b) UMAP plots representing the temporal changes in gene expression in lung endothelium during normal (red-coloured cells) and aberrant (blue-coloured cells) lung development. c) Heatmaps depicting ligand activity affecting endothelial cells: Grey-red colormap corresponds to whether a ligand was in the top 10 ligands predicted to affect the specific cell type (colored red); Purple heatmaps: Colormap represents average log(TP10k+1) expression values of ligands for each cell type in the hyperoxia samples (depicted in violet); Blue-red colormap represents the log(fold change) expression of ligands in hyperoxia samples (depicted in red/blue). d) Heatmaps of receptor activity showing the putative receptors for each ligand. Green colormap represents the prior interaction potential in NicheNet's model (depicted in green); Purple colormap represents average log(TP10k+1) expression values of receptors for each cell type (depicted in violet). e) Dotplot displaying expression patterns of arterial and vein-specific genes in diverse populations within lung endothelium. The intensity of expression in the Dotplot is indicated by the color legend. Size of the cell population expressing the gene of interest is indicated by the size of the circle as specified by the legend. Expression values represent Z-score-transformed log(TP10k+1) values. f) UMAP plots depicting expression of *Kit* in normally and aberrantly developing lungs. The intensity of expression is indicated by purple coloring. Violin plots depicting expression of *Kit1* in clusters 0 and 1 in normally (21% O2, green) and aberrantly (21% O2, red) developing lungs. g) UMAP and violin plots depicting expression of *Procr* and *Bst1* in normally and aberrantly developing lungs at P14. The intensity of expression in UMAP is indicated by purple coloring. Violin plots depicting expression of *Procr* and *Bst1* in clusters 3 and 4 in normally (21% O2, green) and aberrantly (21% O2, red) developing lungs at P14. Expression values in violin plots represent Z-score-transformed log(TP10k+1) values. Expression levels in UMAP plots and Dotplots are presented as log(TP10k+1) values. Log(TP10k+1) corresponds to log-transformed UMIs per 10k.

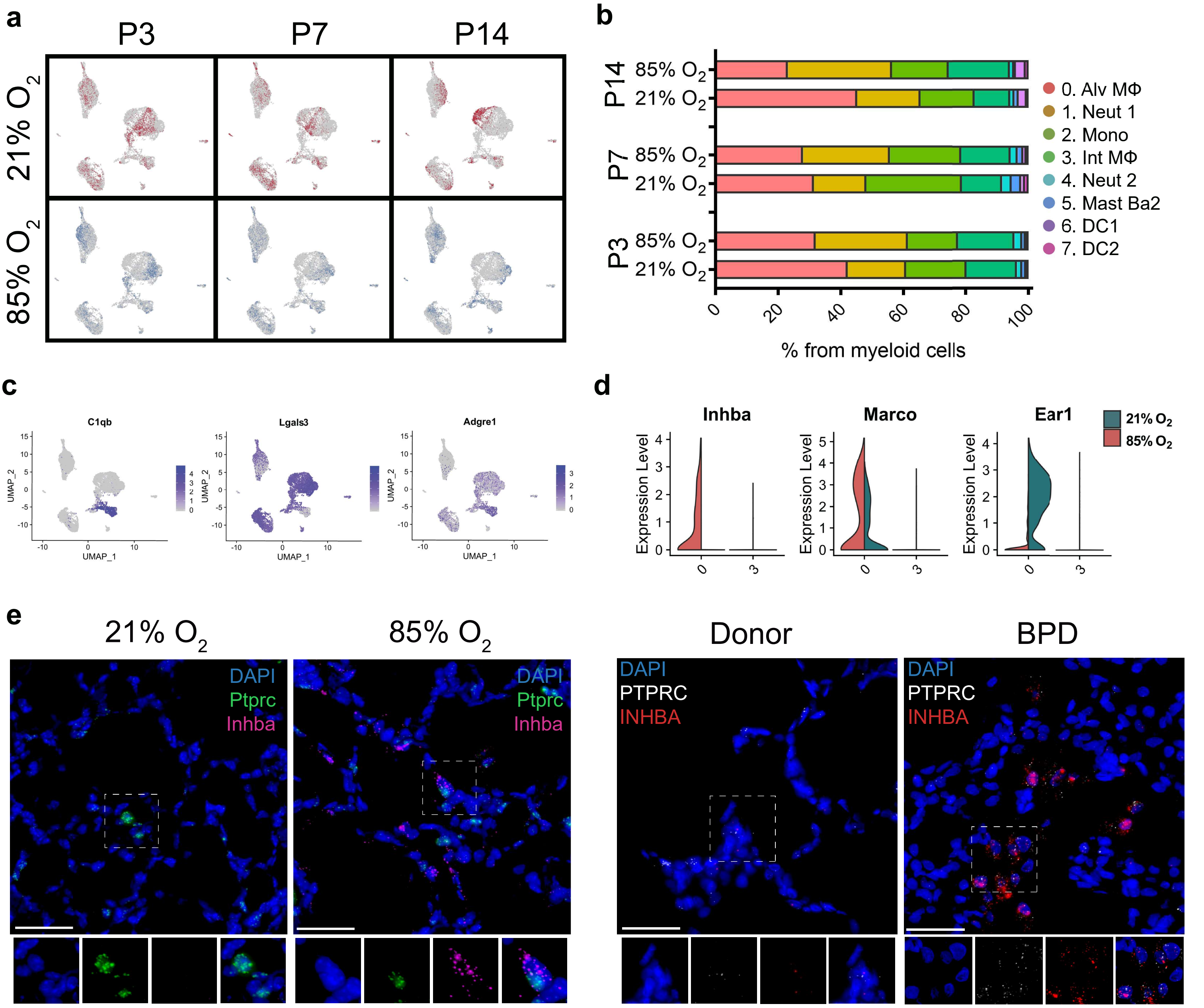

Supplementary figure 8. Cellular composition of normal and hyperoxia-impaired developing lung myeloid populations.

a) UMAP plots representing the temporal changes in gene expression in lung myeloid cells during normal (red-coloured cells) and aberrant (blue-coloured cells) lung development. b) Relative contribution of individual clusters changed significantly during the development and after exposure to hyperoxia. Cell populations are colored as indicated by the legend. n = 6 animals/group c) UMAP plots depicting expression of markers of alveolar and interstitial macrophage populations in normally and aberrantly developing lungs. The intensity of expression is indicated by purple coloring. d) Violin plots depicting the oxygen-specific expression of *Inhba*, *Marco*, *Ear1* in lung macrophages at P14. e) Fluorescent RNA *in situ* hybridization showing coexpression of *Inhba*/INHBA (pink/red) with *Ptprc*/PTPRC (green/white) positive leukocytes morphologically resembling alveolar macrophages in normal and mouse hyperoxic/human BPD lungs, respectively. Magnification: 40x. Scale bar = 40µm. Two 14-days old animals/group were analysed and samples from five BPD patients and two donor lungs were analyzed. Expression values in violin plots represent Z-score-transformed log(TP10k+1) values. Expression levels in UMAP plots are presented as log(TP10k+1) values. Log(TP10k+1) corresponds to log-transformed UMIs per 10k.

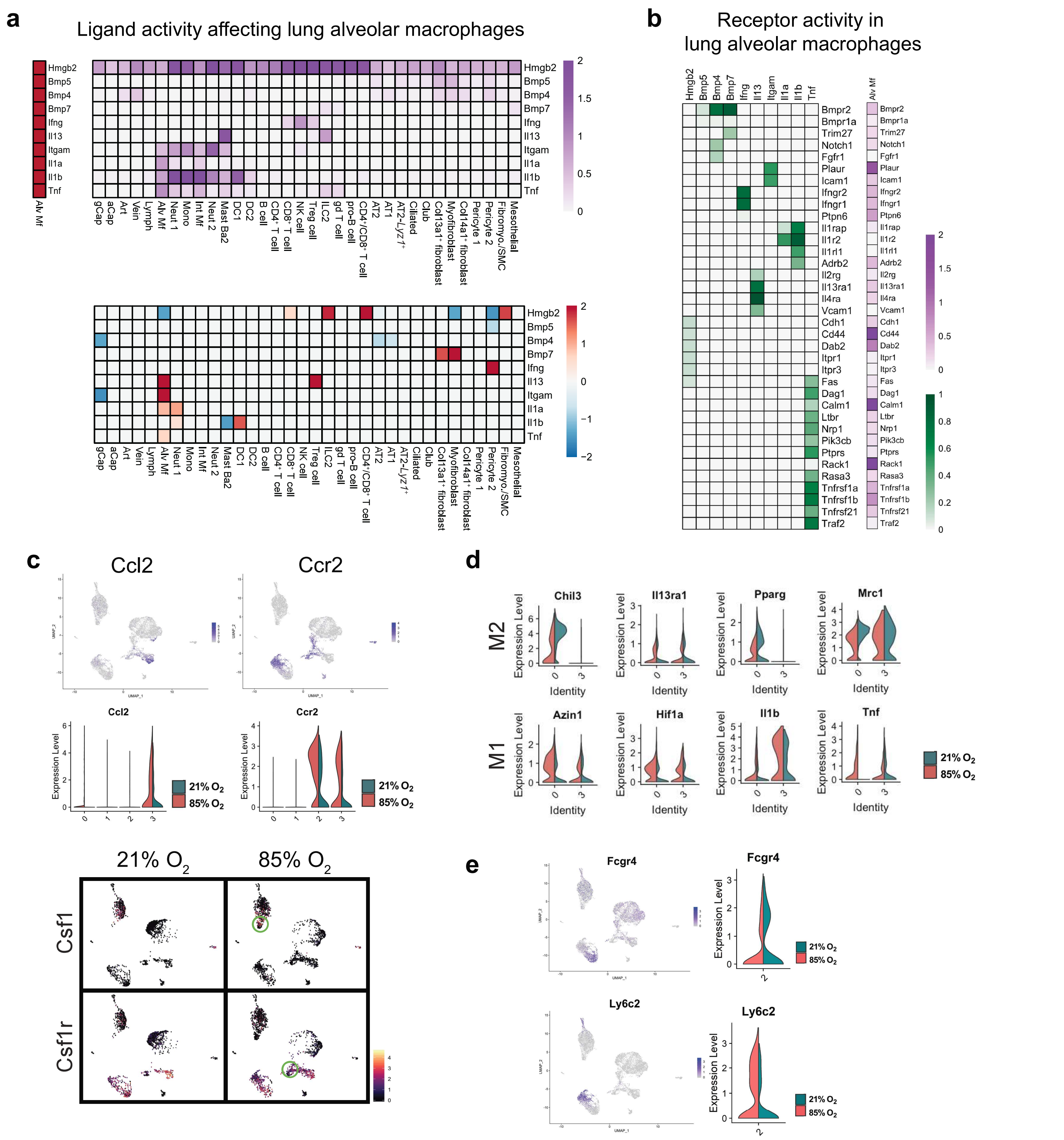

Supplementary figure 9. Cellular communication affecting developing lung myeloid populations.
a) Heatmaps depicting ligand activity affecting myeloid cells: Grey-red colormap corresponds to whether a ligand was in the top 10 ligands predicted to affect the specific cell type (colored red); Purple heatmaps: Colormap represents average log(TP10k+1) expression values of ligands for each cell type in the hyperoxia samples (depicted in violet); Blue-red colormap represents the log(fold change) expression of ligands in hyperoxia samples (depicted in red/blue). b) Heatmaps of receptor activity showing the putative receptors for each ligand. Green colormap represents the prior interaction potential in NicheNet's model (depicted in green); Purple colormap represents average log(TP10k+1) expression values of receptors for each cell type (depicted in violet). c) UMAP plots and violin plots depicting the expression patterns of expression of *Csf1* and *Csf1r* and expression patterns of *Ccr2* and its ligand *Ccl2* at P14 in normally and aberrantly developing lungs. The intensity of expression is indicated by the color legend. d) Violin plots displaying expression patterns of M1 and M2 macrophages-specific markers in the two macrophage populations identified in the study at P14. The intensity of expression is indicated by the color legend. Size of the cell population expressing the gene of interest is indicated by the size of the circle as specified by the legend. e) UMAP plots showing the expression patterns of classical and non-classical monocytes markers in all myeloid cells. The intensity of expression is indicated by purple coloring. Violin plots showing the oxygen-specific expression patterns of classical and non-classical monocytes markers at P14. Expression values in violin plots represent Z-score-transformed log(TP10k+1) values. Expression levels in UMAP plots are presented as log(TP10k+1) values. Log(TP10k+1) corresponds to log-transformed UMIs per 10k.

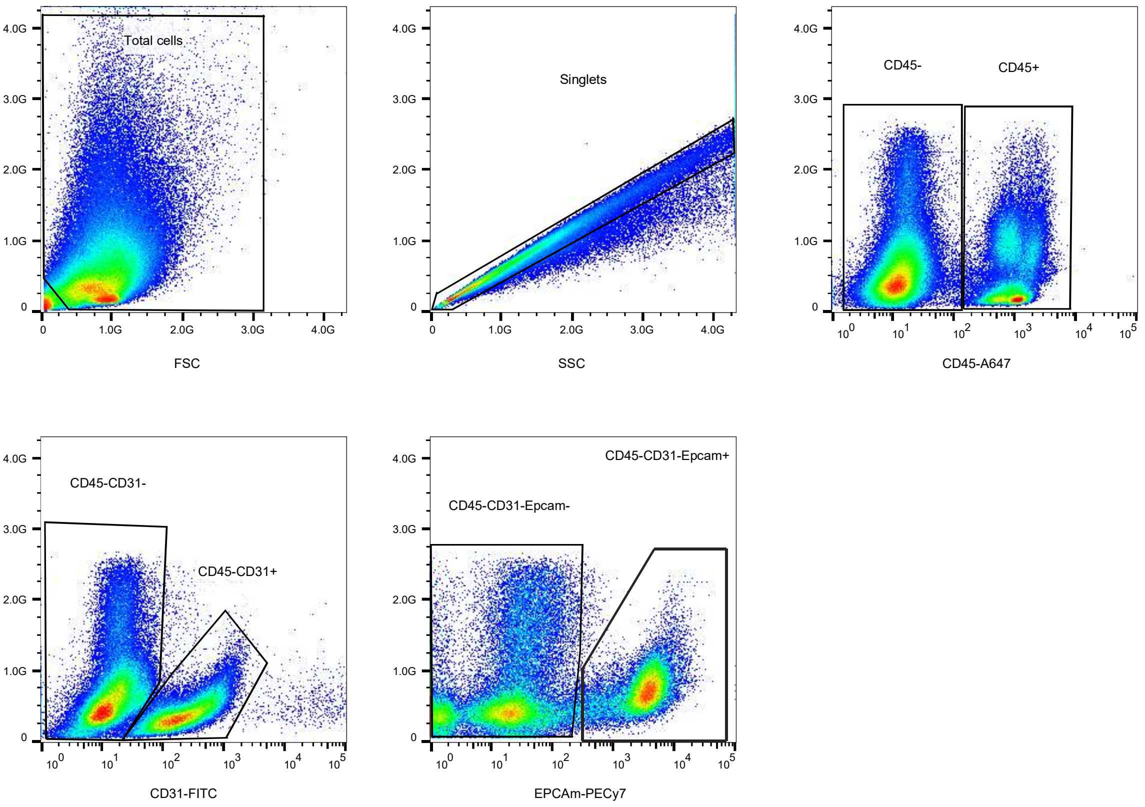

Supplementary fig. 10. Gating strategy.

Immune cells were defined as CD45-AF647^{+}; endothelial cells were defined as CD45-AF647^{-}/CD31-FITC^{+} cells; epithelial cells were defined as CD45-AF647^{-}/CD31-FITC^{-}/CD326-PeCy7^{+} cells; stromal cells were identified as CD45-AF647^{-}/CD31-FITC^{-}/CD326-PeCy7^{-} cells.

Supplementary table 1. Alveolar type 2 cell stereology.

	Normoxia				Hyperoxia			
Lung	1	2	3	4	1	2	3	4
Sv (cm^{-1})	987.97	881.17	884.95	819.39	606.16	573.86	562.15	672.34
Parenchymal fraction	0.89	0.94	0.92	0.92	0.88	0.85	0.93	0.80
Lung Volume (cm^3)	0.158	0.194	0.202	0.209	0.16	0.152	0.145	0.146
Surface Area (cm^2)	138.17	161.38	164.95	157.80	85.22	74.50	76.01	78.80
total AT2:	191	141	139	111	108	76	62	100
total AT2/lung:	3820000	2820000	2780000	2220000	2160000	1520000	1240000	2000000
AT2/surface area	27646.38	17474.25	16853.58	14068.26	25347.37	20402.16	16314.15	25379.51

AT2, alveolar type 2 cell; *Sv*, surface density

Supplementary table 2. Primer sequences.

Gene	Primer orientation	Primer sequence, (5' - 3')	Primer size, bp	Amplicon size, bp
Sry	Forward	TGGGACTGGTGACAATTGTC	20	402
	Reverse	GAGTACAGGTGTGCAGCTCT	20	
Il3	Forward	GGGACTCCAAGCTTCAATCA	20	544
	Reverse	TGGAGGAGGAAGAAAAGCAA	20	

bp, base pair
