# Supplementary material for: Single cell transcriptomic analysis of murine lung development on hyperoxia-induced damage
Source: Nat Commun. 2021 Mar 10;12:1565. doi: 10.1038/s41467-021-21865-2 (PMC7946947; doi:10.1038/s41467-021-21865-2)
Supplement: Supplementary file 2 — Description of Additional Supplementary Files [file 41467_2021_21865_MOESM2_ESM.pdf]

Title: **Supplementary Movie 1. Cellular composition in late lung development.**

Description: A 3D animated scatterplot of all scRNA_seq data, showing a total of 34 distinct cell types that were identified.

Title: **Supplementary Data 1. Cell cluster markers.**

Description: List of cell clusters markers based on most differentially expressed genes.

Title: **Supplementary Data 2. Percentual clusters contribution.**

Description: Tables depict absolute number of cells in each cluster, as well as the percentual contribution of each cluster from total number of sequenced cells. Data correspond to the Supplementary figure 2a.

Title: **Supplementary Data 3. Epithelial subset markers.**

Description: List of epithelial cell clusters markers based on most differentially expressed genes. Differential gene expression for each cell type, calculated using generalized linear models implemented in the R packages muscat and edgeR. False discovery rate-adjusted p-values are included.

Title: **Supplementary Data 4. Hyperoxia-induced changes in gene expression in lung epithelium.**

Description: List of differentially expressed genes in hyperoxia in lung epithelium. Each sheet corresponds to one epithelial cluster. Differential gene expression for each cell type, calculated using generalized linear models implemented in the R packages muscat and edgeR. False discovery rate-adjusted p-values are included.

Title: **Supplementary Data 5. Functional pathway analysis in lung epithelial cells.**

Description: List of differentially regulated functional pathways in hyperoxia in lung epithelium. Each sheet corresponds to one epithelial cluster. Differential gene expression for each cell type,

calculated using generalized linear models implemented in the R packages muscat and edgeR. False discovery rate-adjusted p-values are included.

Title: **Supplementary Data 6. Stroma subset markers.**

Description: List of stromal cell clusters markers based on most differentially expressed genes. Differential gene expression for each cell type, calculated using generalized linear models implemented in the R packages muscat and edgeR. False discovery rate-adjusted p-values are included.

Title: **Supplementary Data 7. Hyperoxia-induced changes in gene expression in lung stroma.**

Description: List of differentially expressed genes in hyperoxia in lung stroma. Each sheet corresponds to one stromal cluster. Differential gene expression for each cell type, calculated using generalized linear models implemented in the R packages muscat and edgeR. False discovery rate-adjusted p-values are included.

Title: **Supplementary Data 8. Functional pathway analysis in lung stromal cells.**

Description: List of differentially regulated functional pathways in hyperoxia in lung stroma. Each sheet corresponds to one stromal cluster. Differential gene expression for each cell type, calculated using generalized linear models implemented in the R packages muscat and edgeR. False discovery rate-adjusted p-values are included.

Title: **Supplementary Data 9. Endothelial subset markers.**

Description: List of endothelial cell clusters markers based on most differentially expressed genes. Differential gene expression for each cell type, calculated using generalized linear models implemented in the R packages muscat and edgeR. False discovery rate-adjusted p-values are included.

Title: **Supplementary Data 10. Hyperoxia-induced changes in gene expression in lung endothelium.**

Description: List of differentially expressed genes in hyperoxia in lung endothelium. Each sheet corresponds to one endothelial cluster. Differential gene expression for each cell type, calculated using generalized linear models implemented in the R packages muscat and edgeR. False discovery rate-adjusted p-values are included.

Title: **Supplementary Data 11. Functional pathway analysis in lung endothelial cells.**

Description: List of differentially regulated functional pathways in hyperoxia in lung endothelium. Each sheet corresponds to one endothelial cluster. Differential gene expression for each cell type, calculated using generalized linear models implemented in the R packages muscat and edgeR. False discovery rate-adjusted p-values are included.

Title: **Supplementary Data 12. Myeloid subset markers.**

Description: List of myeloid cell clusters markers based on most differentially expressed genes. Differential gene expression for each cell type, calculated using generalized linear models implemented in the R packages muscat and edgeR. False discovery rate-adjusted p-values are included.

Title: **Supplementary Data 13. Hyperoxia-induced changes in gene expression in lung myeloid populations.**

Description: List of differentially expressed genes in hyperoxia in lung myeloid populations. Each sheet corresponds to one myeloid cluster. Differential gene expression for each cell type, calculated using generalized linear models implemented in the R packages muscat and edgeR. False discovery rate-adjusted p-values are included.

Title: **Supplementary Data 14. Functional pathway analysis in lung myeloid cells.**

Description: List of differentially regulated functional pathways in hyperoxia in lung myeloid populations. Each sheet corresponds to one myeloid cluster. Differential gene expression for each cell type, calculated using generalized linear models implemented in the R packages muscat and edgeR. False discovery rate-adjusted p-values are included.

Title: **Supplementary Data 15. Lymphoid subset markers.**

Description: List of lymphoid cell clusters markers based on most differentially expressed genes. Differential gene expression for each cell type, calculated using generalized linear models implemented in the R packages muscat and edgeR. False discovery rate-adjusted p-values are included.

Title: **Supplementary Data 16. Hyperoxia-induced changes in gene expression in lung lymphoid populations.**

Description: List of differentially expressed genes in hyperoxia in lung lymphoid populations. Each sheet corresponds to one lymphoid cluster. Differential gene expression for each cell type, calculated using generalized linear models implemented in the R packages muscat and edgeR. False discovery rate-adjusted p-values are included.

Title: **Supplementary Data 17. Functional pathway analysis in lung lymphoid cells.**

Description: List of differentially regulated functional pathways in hyperoxia in lung lymphoid populations. Each sheet corresponds to one lymphoid cluster. Differential gene expression for each cell type, calculated using generalized linear models implemented in the R packages muscat and edgeR. False discovery rate-adjusted p-values are included.

Title: **Supplementary Data 18. Mesothelial subset markers.**

Description: List of mesothelial cell cluster markers based on most differentially expressed genes. Differential gene expression for each cell type, calculated using generalized linear models implemented in the R packages muscat and edgeR. False discovery rate-adjusted p-values are included.

Title: **Supplementary Data 19. Hyperoxia-induced changes in gene expression in lung mesothelial population.**

Description: List of differentially expressed genes in hyperoxia in lung mesothelial populations. Differential gene expression for each cell type, calculated using generalized linear models implemented in the R packages muscat and edgeR. False discovery rate-adjusted p-values are included.

Title: **Supplementary Data 20. Functional pathway analysis in lung mesothelial cells.**

t of differentially regulated functional pathways in hyperoxia in lung mesothelial population. Differential gene expression for each cell type, calculated using generalized linear models implemented in the R packages muscat and edgeR. False discovery rate-adjusted p-values are included.

Title: **Supplementary Data 21. Human tissues.**

Description: Detail information on human BPD and donor sample. *BPD*, bronchopulmonary dysplasia; *CLD*, chronic lung disease ; *ClinPathDx* , Clinical Pathology Diagnosis; *GA*, gestational age; *IVH*, intraventricular hemorrhage ; *NEC*, necrotizing enterocolitis, ; *NI* = normal; *PA*, pulmonary artery; *PHTN*, pulmonary hypertension; *RDS*, respiratory distress syndrome; *RLL*, right lower lobe; *ROP*, retinopathy of prematurity; *RSV*, respiratory syncytial virus; *SGA*, small for gestational age; *UNK*, unknown.
