# Supplementary figures and images for: Single cell transcriptomic analysis of murine lung development on hyperoxia-induced damage
Source: Nat Commun. 2021 Mar 10;12:1565. doi: 10.1038/s41467-021-21865-2 (PMC7946947; doi:10.1038/s41467-021-21865-2)

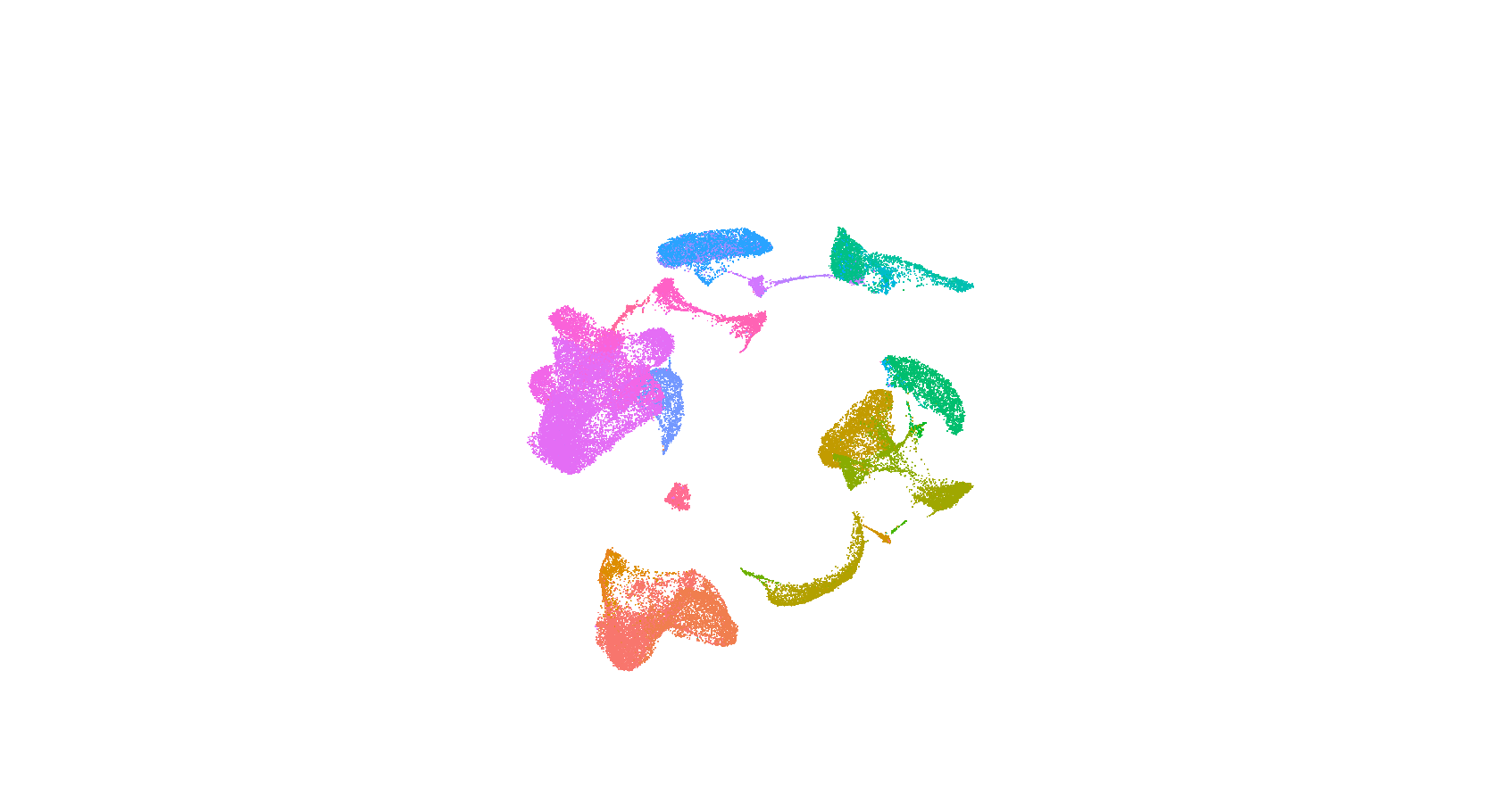

Supplement: Supplementary file 3 — Supplementary Movie 1 [file 41467_2021_21865_MOESM3_ESM.gif]
