# Supplementary material for: Single cell transcriptomic analysis of murine lung development on hyperoxia-induced damage
Source: Nat Commun. 2021 Mar 10;12:1565. doi: 10.1038/s41467-021-21865-2 (PMC7946947; doi:10.1038/s41467-021-21865-2)
Supplement: Supplementary file 25 — Reporting Summary [file 41467_2021_21865_MOESM25_ESM.pdf]

nature research

Corresponding author(s): Bernard Thébaud

Last updated by author(s): Jan 30, 2021

Reporting Summary

Nature Research wishes to improve the reproducibility of the work that we publish. This form provides structure for consistency and transparency in reporting. For further information on Nature Research policies, see our Editorial Policies and the Editorial Policy Checklist.

Statistics

For all statistical analyses, confirm that the following items are present in the figure legend, table legend, main text, or Methods section.

n/a	Confirmed	
☐	☒	The exact sample size (*n*) for each experimental group/condition, given as a discrete number and unit of measurement
☐	☒	A statement on whether measurements were taken from distinct samples or whether the same sample was measured repeatedly
☐	☒	The statistical test(s) used AND whether they are one- or two-sided<br>*Only common tests should be described solely by name; describe more complex techniques in the Methods section.*
☒	☐	A description of all covariates tested
☐	☒	A description of any assumptions or corrections, such as tests of normality and adjustment for multiple comparisons
☐	☒	A full description of the statistical parameters including central tendency (e.g. means) or other basic estimates (e.g. regression coefficient) AND variation (e.g. standard deviation) or associated estimates of uncertainty (e.g. confidence intervals)
☐	☒	For null hypothesis testing, the test statistic (e.g. *F*, *t*, *r*) with confidence intervals, effect sizes, degrees of freedom and *P* value noted<br>*Give P values as exact values whenever suitable.*
☒	☐	For Bayesian analysis, information on the choice of priors and Markov chain Monte Carlo settings
☒	☐	For hierarchical and complex designs, identification of the appropriate level for tests and full reporting of outcomes
☒	☐	Estimates of effect sizes (e.g. Cohen's *d*, Pearson's *r*), indicating how they were calculated

Our web collection on statistics for biologists contains articles on many of the points above.

Software and code

Policy information about availability of computer code

Data collection	No software was used for data collection
Data analysis	Raw sequencing data was processed using CellRanger v3.0.2.<br>scRNA-seq analysis was primarily performed using the R package Seurat v3.1.5.<br>Other software packages include:<br>fgsea v1.12.0<br>deMULTIplex v1.0.2<br>Trimmomatic v0.36<br>Mean linear intercept was assessed using the Quorum Analysis software.<br>Number of AT2 cells in lung sections was assessed using the Stereo Investigator® software.<br>FACS analysis were performed using the FlowJo v10 software.

For manuscripts utilizing custom algorithms or software that are central to the research but not yet described in published literature, software must be made available to editors and reviewers. We strongly encourage code deposition in a community repository (e.g. GitHub). See the Nature Research guidelines for submitting code & software for further information.

Data

Policy information about availability of data

All manuscripts must include a data availability statement. This statement should provide the following information, where applicable:

- Accession codes, unique identifiers, or web links for publicly available datasets
- A list of figures that have associated raw data
- A description of any restrictions on data availability

Raw fastq sequencing files, gene expression matrices, and associated cell metadata have been made available on the NCBI's Gene Expression Omnibus (GEO) at accession number GSE151974. Code used for the analysis of scRNA-seq data is available at the public GitHub repository at https://github.com/dpcook/thebaud_lung_BPD.

Field-specific reporting

Please select the one below that is the best fit for your research. If you are not sure, read the appropriate sections before making your selection.

☒ Life sciences ☐ Behavioural & social sciences ☐ Ecological, evolutionary & environmental sciences

For a reference copy of the document with all sections, see nature.com/documents/nr-reporting-summary-flat.pdf

Life sciences study design

All studies must disclose on these points even when the disclosure is negative.

Sample size	Previously published studies on scRNA-seq analysis in postnatal developing mice lungs have included no more than 3 replicates Guo et al., Nat. Comm., 2019; Cohen et al., Cell, 2018 (each replicate being individual mouse). In order to improve the reproducibility we chose to analyze 6 animals /group, which exceeds the current standard in the field.
Data exclusions	Due to exploratory nature of scRNA-seq study, no data were excluded from statistical analysis.
Replication	Lungs from individual mice within each group were isolated at two different occasions and sequenced separately to adjust for any bias and to explore the reproducibility. As seen from Supplemental figure 2a, individual values cluster closely together indicating good reproducibility.<br>All performed experiments are included and we did not exclude any experiments from our analysis.
Randomization	All mice born on the same day were randomized - assigned randomly to a nursing dam - prior to assignment to an experimental group.<br>The human tissue collection of these rare patients was not randomized and was organized by the Biorepository for Investigation of Neonatal Diseases of Lung-Normal (BRINDL-NL).
Blinding	In case of FACS analysis and lung histology analysis, researchers were blinded to group allocation, data collection and data analysis. In case of scRNA-seq, researcher performing the sequencing was blinded to group allocation and data collection. Data analysis of scRNA-seq was not blinded, as the analysis is entirely data driven and therefore unbiased. For the single molecule in situ hybridization experiments in mice tissues, both the investigator performing the staining and doing the analysis were blinded for the group allocation, data collection and data analysis. However, due to the very distinct patho-histology seen in human BPD lung samples (obvious for the investigator), blinding was not used for quantification of INHBA in situ hybridization experiments in human lung samples.

Reporting for specific materials, systems and methods

We require information from authors about some types of materials, experimental systems and methods used in many studies. Here, indicate whether each material, system or method listed is relevant to your study. If you are not sure if a list item applies to your research, read the appropriate section before selecting a response.

Materials & experimental systems

n/a	Involved in the study	
☐	☒	Antibodies
☒	☐	Eukaryotic cell lines
☒	☐	Palaeontology and archaeology
☐	☒	Animals and other organisms
☐	☒	Human research participants
☒	☐	Clinical data
☒	☐	Dual use research of concern

Methods

n/a	Involved in the study	
☒	☐	ChIP-seq
☐	☒	Flow cytometry
☒	☐	MRI-based neuroimaging

Antibodies

Antibodies used	For HC staining: proSP-C using an anti-proSP-C antibody (AB3786; Millipore/Sigma, Entobicoke, ON, Canada).

For FACS: FITC-conjugated CD31 (558738; BD Biosciences, Mississauga, ON, Canada); AF647-conjugated CD45 (1660-31; Southern Biotech, Birmingham, AL, USA); Pe/Cy7-conjugated CD326 (EpCAM; 25-5791-80 / 501129753; Thermofischer Scientific, Burlington, ON, Canada); CD16/CD32 FcBlock (553142, BD Biosciences, Mississauga, ON, Canada).

Validation

The proSP-C antibody was validated for ELISA, immunohistochemistry (IHC), and western blotting by Millipore Sigma. Reactive for human, mouse and rat. Recommended use for IHC is 1:2000 to 1:4000 in human lung tissue, 1:1000 to 1:2000 in adult and 1:500 to 1:1000 in fetal mouse lung.

The CD31-FITC (BDBiosciences), CD45-AF647 (Southern Biotech), and CD326/EpCAM-PE-Cy7 (eBiosciences) antibodies were validated for flow cytometry by the manufacturers. All 3 antibodies have their lots quality control tested by immunofluorescent staining with flow cytometry analysis. All 3 antibodies are reactive for mouse.

Animals and other organisms

Policy information about studies involving animals; ARRIVE guidelines recommended for reporting animal research

Laboratory animals	Mice strain used was C57BL/6N. Developing pups were 3, 7 or 14 days old on the day of harvest. Animals of both sexes were represented. The animal experiments were performed at the University of Ottawa animal facility where the animals were on a 12 h on, 12 h off light cycle, room temperature was 21°C, and humidity was 45%. These are all within the range of guidelines set by the Canadian Council on Animal Care.
Wild animals	No wild animals were used in the study.
Field-collected samples	No field-collected samples were used in the study.
Ethics oversight	All animal procedures were approved by the Animal Care Committee of the University of Ottawa under animal ethics protocol OHRI-1696.

Note that full information on the approval of the study protocol must also be provided in the manuscript.

Human research participants

Policy information about studies involving human research participants

Population characteristics	Paraffin-embedded lung sections from BPD patients and age-matched donors were kindly provided by the LungMAP Human Tissue Core, Biorepository for Investigation of Neonatal Diseases of Lung-Normal (BRINDL-NL). Detailed information about all samples is provided in Supplementary table 23.
Recruitment	Written consent was provided by parents/guardians of minor participators.
Ethics oversight	University of Rochester, Rochester, NY 14627-0140

Note that full information on the approval of the study protocol must also be provided in the manuscript.

Flow Cytometry

Plots

Confirm that:

- [x] The axis labels state the marker and fluorochrome used (e.g. CD4-FITC).
- [x] The axis scales are clearly visible. Include numbers along axes only for bottom left plot of group (a 'group' is an analysis of identical markers).
- [x] All plots are contour plots with outliers or pseudocolor plots.
- [x] A numerical value for number of cells or percentage (with statistics) is provided.

Methodology

Sample preparation

Single-cell suspensions were obtained form lung homogenates from developing mice. Lung tissue was digested using following enzymatic mixture: 2500U Collagenase I (Wothington Biochem., Lakewood, NJ, USA), 30U Neutral Protease (Wothington Biochem., Lakewood, NJ, USA), 500U Deoxyribonuclease (DNAse) I (Sigma-Aldrich, Oakville, ON, Canada) and homogenized at 37°C by gentleMACS™ Octo Dissociator (Miltenyi Biotech, Bergisch Gladbach, Germany).
The number of cells in single-cell suspension was estimated using a Scepter™ automated cell counter (Millipore-Sigma, Burlington, MA, USA) and total of 0.5×106 cells/sample were resuspended in 200 µl of PBS in 96-well plate. Cells were incubated in the dark with 2 µl/1×106 cells of CD16/32 antibody (Fc block; BD Biosciences, Mississauga, ON, Canada) for 15 minutes at RT. Following blocking, cells were centrifuged and resulting pellets were resuspended in 1:100 mixture of panel of antibodies: FITC-conjugated CD31 (BD Biosciences, Mississauga, ON, Canada), AF647-conjugated CD45 (Southern Biotech, Birmingham, AL, USA), Pe/Cy7-conjugated CD326 (EpCAM; Thermofischer Scientific, Burlington, ON, Canada), Pe-conjugated CD144 (VE-Cadherine; BD Biosciences, Mississauga, ON, Canada). Cells were incubated with antibodies at RT for 30 minutes in dark. Following staining, cells were pelleted by centrifugation and washed 3x with FACS buffer (5% (v/v) FBS and 1mM EDTA in 1×DPBS). All samples were fixed by 4% (w/v) PFA prior to analysis.

Instrument	The BeckmanCoulter MoFlo XDP (Beckman Coulter Life Sciences, Indianapolis, IN, USA)
Software	FlowJo v10 software (FlowJo LLC, Ashland, OR, USA)
Cell population abundance	N/A
Gating strategy	Immune cells were defined as CD45-AF647+; endothelial cells were defined as CD45-AF647-/CD31-FITC+ cells; epithelial cells were defined as CD45-AF647-/CD31-FITC-/CD326-PeCy7+ cells; stromal cells were identified as CD45-AF647-/CD31-FITC-/CD326-PeCy7- cells.

☒ Tick this box to confirm that a figure exemplifying the gating strategy is provided in the Supplementary Information.
